# Functional and evolutionary characteristics of human genes encoding cell surface receptors involved in the regulation of appetite

**DOI:** 10.1515/jib-2025-0023

**Published:** 2026-01-08

**Authors:** Elena Ignatieva, Sergey Lashin, Roman Ivanov, Valentin Suslov, Angelina Mikhailova, Nikolay Kolchanov

**Affiliations:** Institute of Cytology and Genetics, SB RAS, Novosibirsk, Russia; Novosibirsk State University, Novosibirsk, Russia

**Keywords:** development, divergence index, food intake, GPCR superfamily, phylostratigraphic age

## Abstract

Appetite is an instinct that has been formed through evolution. Appetite promotes normal growth and development in humans. However, under conditions of food abundance, appetite can become excessive, posing significant health risks. In this study we have identified 80 human genes whose orthologs regulated food intake in model animal species. More than 80 % of these genes encode G-protein-coupled receptors and 29 % were found to be involved in developmental processes. Using phylostratigraphic age index (PAI), which specifies the evolutionary age of a gene, we found that this set of 80 genes contains an increased proportion of genes with the same phylostratigraphic age (PAI = 6, the stage of Vertebrata divergence) indicating the coordinated evolution of this group of genes. Using divergence index (DI), which indicates the type of selection to which the gene is subjected, we observed significant enrichment for genes with DI ≤ 0.25, i.e., those that are subject to strong stabilizing selection. The subgroup of genes having DI ≤ 0.25 included 45 genes and was enriched with genes that are associated with developmental processes. This finding supports the hypothesis that developmental disturbances generally impose strong constraints on viability due to purifying selection.

## Abbreviations

5-HT1B: 5-hydroxytryptamine receptor 1B
5-HT1D: 5-hydroxytryptamine receptor 1D
ADCYAP1R1: ADCYAP receptor type I
ADIPOR1: adiponectin receptor 1
ADIPOR2: adiponectin receptor 2
AgRP: agouti-related protein
AGTR2: angiotensin II receptor type 2
AVPR1A: arginine vasopressin receptor 1A
BDKRB1: bradykinin receptor B1
BDNF: brain-derived neurotrophic factor
BMP2: bone morphogenetic protein 2
BMP4: bone morphogenetic protein 4
BMP6: bone morphogenetic protein 6
BMPR1A: bone morphogenetic protein receptor type 1A
CALCR: calcitonin receptor
cAMP: cyclic adenosine monophosphate
CCKAR: cholecystokinin A receptor
CCKBR: cholecystokinin B receptor
CMKLR1: chemerin chemokine-like receptor 1
CNR1: cannabinoid receptor 1
CRHR1: corticotropin releasing hormone receptor 1
DAVID: database for annotation, visualization, and integrated discovery
DI: divergence index
DRD1: dopamine receptor D1
DRD2: dopamine receptor D2
EDG1: sphingosine-1-phosphate receptor 1
EDG3: sphingosine-1-phosphate receptor 3
EPH: ephrins
EPHA3: EPH receptor A3
ERK1/2: extracellular signal-regulated kinase 1/2
ESR1: estrogen receptor 1
FFAR3: free fatty acid receptor 3
GABA: gamma-aminobutyric acid
GABAB2: gamma-aminobutyric acid type B receptor subunit 2
GFRAL: GDNF family receptor alpha like
GHR: growth hormone receptor
GHSR: growth hormone secretagogue receptor
GLP2R: glucagon like peptide 2 receptor
GO: Gene Ontology
GPBAR1: G protein-coupled bile acid receptor 1
GPCR: G-protein-coupled receptors
GPCRdb: G protein-coupled receptors database
GPR171: G protein-coupled receptor 171
GPR26: G protein-coupled receptor 26
GPR39: G protein-coupled receptor 39
GPR75: G protein-coupled receptor 75
HEK293: human embryonic kidney 293 cells
HSCs: hematopoietic stem cells
HTR1A: 5-hydroxytryptamine receptor 1A
HTR2C: 5-hydroxytryptamine receptor 2C
IL1R1: interleukin 1 receptor type 1
Insl5: insulin-like peptide 5
INSR: insulin receptor
KEGG SSDB: Kyoto encyclopedia of genes and genomes sequence similarity dataBase
KISS1R: kisspeptin-1 metastasis suppressor receptor
LCA: last common ancestor
LEPR: leptin receptor
LGR4: leucine rich repeat containing G protein-coupled receptor 4
LRP1: LDL receptor related protein 1
LRP6: LDL receptor-related protein 6
MC1R: melanocortin 1 receptor
MC3R: melanocortin 3 receptor
MC4R: melanocortin 4 receptor
MCHR1: melanin concentrating hormone receptor 1
NFKB: nuclear factor kappa-light-chain-enhancer of activated B cells
NPBWR1: neuropeptides B and W receptor 1
NPY: neuropeptide Y
NPY1R: neuropeptide Y receptor 1
NPY2R: neuropeptide Y receptor 2
NPY4R: neuropeptide Y receptor 4
NPY5R: neuropeptide Y receptor 5
NTRK2: neurotrophic receptor tyrosine kinase 2
OPRD1: opioid receptor delta 1
OR51E2: olfactory receptor family 51 subfamily E member 2
OXTR: oxytocin receptor
PAI: phylostratigraphic age index
POMC: proopiomelanocortin
PYY: polypeptide YY
QRFPR: pyroglutamylated RFamide peptide receptor
RXFP4: relaxin family peptide/INSL5 receptor 4
SI: specificity indices
SIGMAR1: sigma non-opioid intracellular receptor 1
Src/Ras-dependent: pathway
TLR2: toll like receptor 2
TLR4: toll like receptor 4
TNF-α: tumor necrosis factor -alph
TSEA: tissue specific expression analysis
UniProt: universal protein knowledgebase
Y5H2B: *C. elegans* gene encoding DUF2250 domain-containing protein
α-MSH: alpha-melanocyte stimulating hormone

## 1 Introduction

Appetite is a physiological mechanism (or the desire of an animal organism to consume food) that regulates the amount of food eaten. The intake of nutrients is necessary for the normal development of any organism. During early ontogeny (prenatally), organisms develop using nutrients derived from the egg or via maternal supply. After birth and throughout life, nutrient intake is regulated by eating behaviour. Eating behaviour is associated with weight gain in infancy and childhood [1]. In Eutheria (placental mammals), the peripheral and central fetal orexic mechanisms develop very early, *in utero*. This is evidenced by the ability of human and other mammalian fetuses to swallow amniotic fluid, and such swallowing is activated after both oral sucrose infusion and central injection of neuropeptide Y, which has orexigenic activity in adult animals [2].

Appetite, or the drive to eat, is inherent in humans and other animal species. The physiological mechanisms regulating appetite have been formed through evolution over a long period. Appetite promotes survival by allowing excessive amounts of food to be consumed during periods of availability in order to prepare for prolonged periods of nutrient deficiency [3]. This physiological mechanism promoted the survival of individuals in populations both in humans and in other animal species. However, this instinct, which gave individuals an advantage in unfavourable conditions, turned out to be excessive for some individuals living in developed countries, where the lifestyle is characterized by an excess of food and insufficient physical activity [3]. This was the reason why obesity has become one of the most serious public health problems of the twenty-first century [4].

The products of genes expressed both in the brain and in various peripheral organs and tissues (intestine, stomach, adipose tissue, and pancreas) provide control over the consumption of food in humans and other animal species [5], [6], [7]. The motivational drive to obtain food is controlled by neurons located in various brain regions such as arcuate, dorsomedial and paraventricular hypothalamic nuclei, amygdala, lateral hypothalamus, raphe magnus/raphe obscurus, ventral tegmental area, nucleus of the solitary tract, prefrontal cortex and a number of others [8]. This system of neurons integrates interoceptive and humoral signals as well as sensory (visual, olfactory and taste) signals [3], 6], 8].

It is assumed that there are at least two main kinds of appetite: homeostatic and non-homeostatic appetite [9]. In conditions of energy and nutrient shortages, food consumption is controlled by homeostatic appetite. However, even with a sufficient amount of food, its smell, taste and sight, as well as the anticipation of pleasant feelings that arise when eating, and other environmental signals can stimulate appetite, which in this case is called non-homeostatic. This type of appetite can induce so-called hedonic feeding aimed at obtaining food reward [10], [11], [12], [13]. These two forms of behaviour are controlled by two different neural systems that function in close cooperation [9], 10].

The central players in the regulation of both homeostatic and non-homeostatic appetite are the neurons of the arcuate nucleus located in the hypothalamus. These neurons secrete alpha-melanocyte stimulating hormone (α-MSH), neuropeptide Y (NPY) and agouti-related protein (AgRP) [3]. The activity of these neurons may be controlled by neurotransmitters (adrenaline, gamma-aminobutyric acid (GABA), serotonin, dopamine), brain-derived neurotrophic factor (BDNF), as well as various hormones (ghrelin, insulin, leptin, peptide YY (PYY), adrenocorticotropin, glucocorticoids, corticotropin-releasing hormone) [3], 8], 14]. The activity of neurons involved in appetite regulation can also be influenced by metabolites. For example, glucose regulates hypothalamic neurons expressing NPY and POMC [15].

Many proteins regulating eating behaviour are also involved in developmental processes. This function is well-established for NTRK2 [16], 17], the receptor for the brain derived neurotrophic factor (BDNF) which is known to cause an anorexic effect [18]. But other receptors involved in the control of eating behaviour, such as GHSR (growth hormone secretagogue receptor), NPY1R (neuropeptide Y receptor 1), NPY2R (neuropeptide Y receptor 2), NPY5R (neuropeptide Y receptor 5) can also mediate the effects of their ligands on development, growth and morphogenesis [19], [20], [21].

In humans, severe disturbances in eating behaviour can be either the cause or consequence of diseases such as bulimia nervosa, obesity and anorexia nervosa. In addition, a number of diseases (neurodegenerative diseases, cancer, chronic autoimmune and inflammatory processes) are accompanied by a decrease in appetite, which in turn increases the severity of these diseases [22]. Given these circumstances, any new knowledge about the system of genes regulating appetite is of particular importance.

Important elements of the molecular-genetic system controlling appetite are cell surface receptors [23], [24], [25], [26]. These receptors interact with the extracellular signalling molecules (neuropeptides, neurotransmitters, releasing factors, hormones, metabolites, etc.) and mediate activation of appetite regulating neurons in various brain regions. Notably that many of the membrane receptors involved in appetite regulation belong to the superfamily of G protein coupled receptors [25], 26]. This finding is consistent with the fact that almost one third of all receptors encoded in the human genome belong to this superfamily [27], 28].

Previously, we performed a functional classification of genes for which data on their role in the control of eating behaviour and body weight were found in scientific publications [23], 24]. It was found that this set (1) was enriched with genes that have brain-specific expression pattern; (2) contained a significant proportion of genes encoding cell surface receptors, in particular, receptors from the superfamily of G protein-coupled receptors (GPCR) [23], 24]. The examples of GPCRs involved in appetite regulation are MC3R (melanocortin 3 receptor), MC4R (melanocortin 4 receptor), GHSR (growth hormone secretagogue receptor), CCKAR (cholecystokinin A receptor), CCKBR (cholecystokinin B receptor) and NPY1R (neuropeptide Y receptor 1) [29].

Understanding the evolutionary mechanisms of human diseases associated with impaired appetite regulation holds fundamental significance and practical importance. It is especially important to carry out such an analysis, since many human diseases (including eating disorders) can be triggered by lifestyle changes that have occurred over the past 100–200 years. It is also very important to investigate the relationship between the evolutionary characteristics of genes involved in a particular network and the sustainability of this network to gene mutations that can affect any gene regions (both coding and regulatory). Integrating phylogenetic and population genetic analysis of genes within regulatory networks may be useful for developing approaches to personalized disease prevention and targeted drug therapy.

Previously, we developed the *Orthoscape* software that allows to evaluate the evolutionary characteristics of genes using phylostratigraphic age index (PAI) and the divergence index (DI) [30], 31]. The PAI estimates the phylostratigraphic age of a gene, and the DI indicates the nature of selection to which the gene is subjected (stabilizing or driving).

The aim of this study is to analyze the functional and evolutionary characteristics of genes encoding cell surface receptors involved in appetite regulation, with special attention to genes associated with the developmental processes.

We have collected a set of human genes encoding cell surface receptors whose orthologs are involved in the regulation of appetite, and identified subgroups of genes with certain functional characteristics (genes encoding GPCRs, genes with brain-specific expression pattern and genes associated with developmental processes). Finally, the evolutionary characteristics of genes were analyzed: the distributions of genes according to the values of the PAI (phylostratigraphic age index) and DI (divergence index) were constructed, and a comparison was made with similar distributions obtained for all human protein-coding genes, as well as for genes encoding GPCRs.

## 2 Materials and methods

### 2.1 Collection of genes involved in appetite regulation and encoding cell surface receptors

The set of genes was obtained from [23] and expanded by searching PubMed for articles published in 2021, 2022, and 2023. The keywords used for the query are listed in Table S1. We restricted our search to experimental studies. In all studies found, the role of genes in the regulation of food intake has been identified using model organisms (e.g. mice, rats, etc.). Therefore, appropriate human orthologs were identified and included into the final list of genes. Evidence that the gene encodes a cell surface receptor was obtained from the text field “Summary” of the EntrezGene database (https://www.ncbi.nlm.nih.gov/gene). The set of genes obtained at this stage is hereinafter referred to as *Receptors_80.*

### 2.2 Control sets of genes

We also compiled additional sets of genes (Table 1). The set *allCDS_19,504* included 19,504 protein-coding genes for which PAI and DI values were identified. The set *allGPCR_420* contained genes encoding GPCRs from the GPCRdb (https://gpcrdb.org), which is a reliable and regularly updated source of information on the receptors from the GPCR superfamily [32], 33]. The list of human genes encoding GPCRs was downloaded 6 March 2024 from the website GPCRdb -> Sequence Analysis -> Receptor Similarity -> Phylogenetic trees. The set *appGPCR_67* comprises genes encoding GPCRs that control appetite. The set *appGPCR_67* was generated by intersecting the *Receptors_80* and *allGPCR_420* sets. The set *app_not_GPCR_13* listed genes encoding receptors that do not belong to the G protein-coupled receptor superfamily. We obtained this set by excluding GPCRs genes from the *Receptors_80* set.

**Table 1:** Gene sets used in the analysis of evolutionary characteristics of genes encoding cell surface receptors and involved in appetite regulation.

Short name	Description	Number of genes
*Receptors_80*	Human protein-coding genes encoding cell surface receptors and involved in appetite regulation^a^.	80
*appGPCR_67*	Genes from the set *Receptors_80* that encode GPCRs	67
*app_not_GPCR_13*	Genes from the set *Receptors_80* encoding receptors that do not belong to the G-protein-coupled receptor superfamily	13
*app_Development_23*	Genes from the *Receptors_80* set that may be involved developmental processes^b^	23
*allCDS_19,504*	All protein-coding genes of the human genome for which PAI and DI values are known.	19,504
*allGPCR_420*	Human genes encoding GPCRs (this set included genes from the GPCRdb (https://gpcrdb.org)).	420

^a^This set includes human genes orthologous to genes of other animal species, the role of which in appetite regulation had been studied experimentally. ^b^According to DAVID these genes were associated with GO terms that contained words *development*, *growth* and *morphogenesis* (see Table S5).

### 2.3 Analysis of tissue-specific characteristics of genes

To identify groups of genes with tissue-specific expression patterns, we used TSEA tool [34]. The TSEA tool (http://doughertytools.wustl.edu/TSEAtool.html) analyzes tissue-specific patterns of gene expression. It operates with data on specificity indices (SI) of gene expression products and their corresponding *p*-values (pSI) calculated for each organ or tissue and for each transcript obtained from the whole transcriptome profiling [35]. Gene expression patterns detected in 25 different human organs and tissues were taken into consideration. We assumed that the gene has tissue-specific expression pattern for a given tissue if its pSI value is <0.01.

### 2.4 Functional annotation of genes using DAVID tool

To identify genes involved in the regulation of biological processes related to development, we used gene annotation by GO terms obtained from DAVID tool [36]. The annotation was carried out using the GOTERM_BP_DIRECT dictionary. We selected genes annotated with terms containing the words *development*, *growth*, and *morphogenesis*.

### 2.5 Classification of receptors by ligand type

For the genes from the GPCR superfamily, we retrieved the type of ligand annotated in the GPCRdb (https://gpcrdb.org). GPCRdb classifies ligands into such types as: nucleotide, peptide, protein, lipid, steroid, ion, amino acid, etc. For other genes the ligand types were determined from the description of receptor function provided by the UniProtKB (https://www.uniprot.org/).

### 2.6 Analysis of the evolutionary characteristics of genes

Evolutionary characteristics of genes were evaluated using phylostratigraphic age index (PAI) and divergence index (DI) which were calculated for all human protein-coding genes as described in [30].

PAI indicates how far from the root of the phylogenetic tree there is a taxon reflecting the age of the gene, i.e., the taxon where the studied species diverged from the most distant related taxon in which the ortholog of the studied gene was found. The values of the PAI were calculated in the *Orthoscape* program based on the KEGG SSDB (Sequence Similarity DataBase), taking into account protein sequences of orthologous genes that are 50 % or more identical to the one under consideration, as described in [30], 31]. PAI ranges from 1 to 16 (Table 2), where “1” corresponds to cellular organisms (the root of the phylogenetic tree), “2” corresponds to the stage of Eukaryota divergence, and “16” corresponds to *Homo sapiens*. Thus, the lower the PAI value, the greater the phylostratigraphic age of the gene (such genes with low PAI are said to be more “ancient”).

**Table 2:** The list of taxons used in phylostratigraphic analysis of *H. sapiens* genes.

PAI value	Taxon	Other alias	Age (Mya^a^)
1	Cellular organism	Cellular organisms	4,100 [37]
2	Eukaryota	Eukaryotes	1,850 [38]
3	Metazoa	Multicellular organisms	665 [39]
4	Chordata	Chordates	541 [40]
5	Craniata	Craniates	535 [40]
6	Vertebrata	Vertebrates	525 [41]
7	Euteleostomi	Bony vertebrates	420 [42]
8	Mammalia	Mammals	225 [43]
9	Eutheria	Placental mammals	160 [44]
10	Euarchontoglires	Supraprimates	65 [45]
11	Primates	Primates	55 [46]
12	Haplorrhini	Monkeys	50 [47]
13	Catarrhini	Catarrhine monkeys	44 [48]
14	Hominidae	Hominids/great apes	17 [49]
15	Homo	Humans	2.8 [50]
16	*Homo sapiens*	Modern humans	0.35 [51]

^a^Million years ago.

Divergence index (DI) indicates the nature of the selection to which the gene is subjected, whether stabilizing or driving. The DI values were calculated as the mean value of the d*N*/d*S* ratio across pairwise comparisons between the human gene and its orthologs in the following closely related hominids: *Pan troglodytes* (chimpanzee), *Pan paniscus* (bonobo), *Gorilla gorilla gorilla* (western lowland gorilla), *Pongo abelii* (Sumatran orangutan)), as described in [30].

The DI values were calculated as the ratio:

$$\text{DI}=\frac{\sum_{i=1}^{n}\text{d}N/\text{d}S_{i}}{n},$$

where d*N* is the proportion of non-synonymous substitutions in the sequences of the studied gene and its orthologue; d*S* is the proportion of synonymous substitutions; *n* is the number of orthologous genes. Based on the DI, we inferred the predominant mode of selection acting on the protein-coding sequence: (1) DI significantly less than 1: strong stabilizing selection; (2) DI ≈ 1: evolution consistent with neutrality; (3) DI significantly greater than 1: evidence for diversifying selection.

Based on the analysis of the values of PAI and DI, we constructed distributions for all protein-coding genes and genes, involved in appetite regulation, as well as other groups of genes presented in Table 1.

### 2.7 Statistical analysis

The numbers of genes with certain characteristics were compared with the expected numbers matching the background distribution for the control gene sets using a chi-square test. Differences between groups were considered statistically significant at *p* < 0.05.

## 3 Results

### 3.1 Human genes encoding cell surface receptors involved in appetite regulation

We have analyzed research articles presenting the results of experimental studies on eating behaviour performed on model organisms (e.g. mice, rats, etc.). These animals were either wild-type or genetically modified (knockout and knockdown) [52], 53], and were subjected to either pharmacological blockade or activation of receptors [54], 55]. Articles with such data were found in PubMed using the following terms: *hyperphagia, hypophagia, bulimia, bulimia nervosa, hyperorexia nervosa, anorexia nervosa, anorexia, eating disorder, food intake, feeding behaviour, appetite, hunger, anorexigenic effect, orexigenic effect, lean, satiety*. These articles contained data on the associations between genes and manifestations of appetite (mainly changes in the amount of food consumed). For further analysis, we formed a final set that included 80 human genes (Table S2), orthologous to genes found in model organisms. Hereafter, this set will be referred to as *Receptors_80*.

### 3.2 Functional characteristics of genes encoding receptors involved in appetite regulation

**Figure 1:**
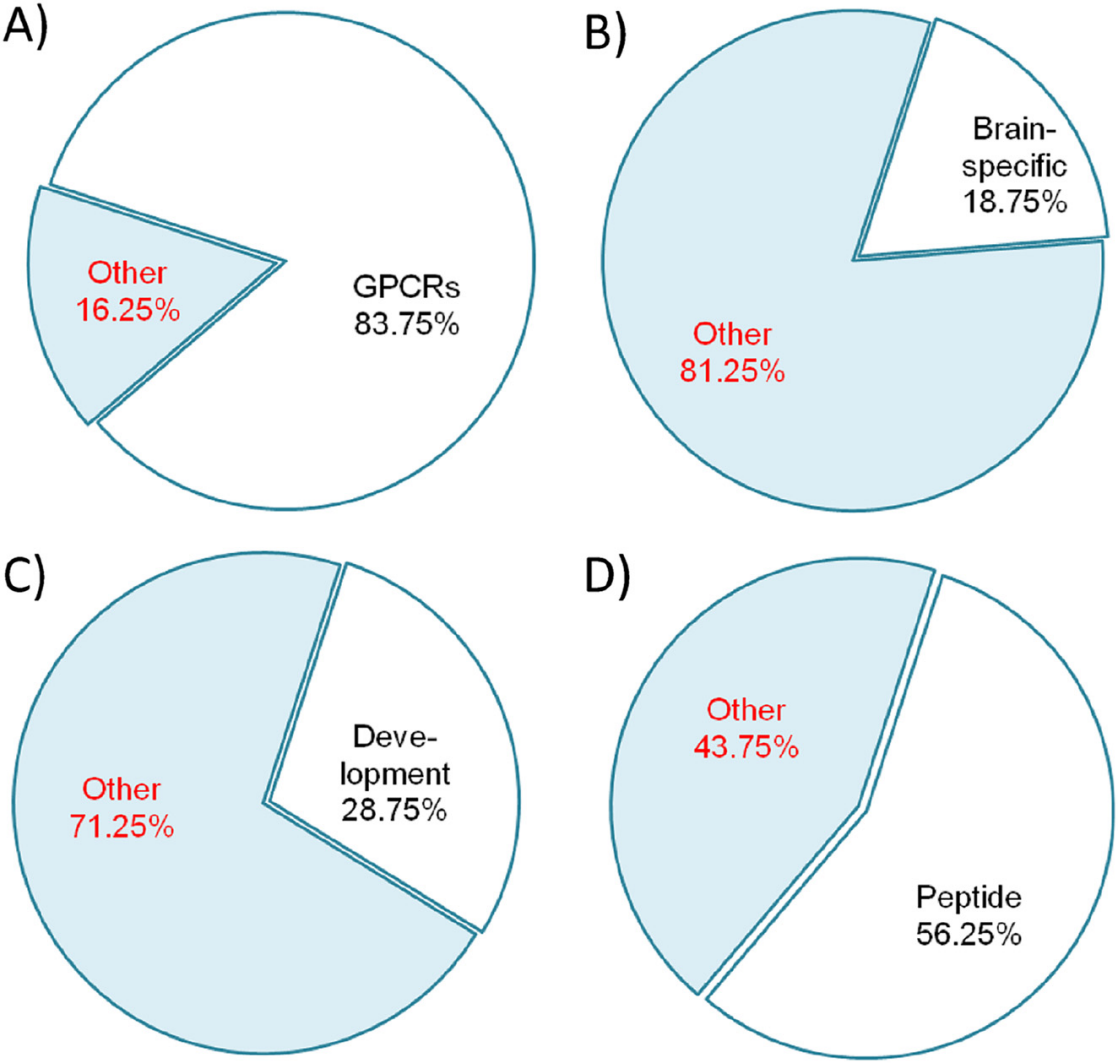
Functional groups of genes identified within the *Receptors_80* set. Panel A – the proportion of genes encoding GPCRs account for ∼84 percent of the total. Panel B – genes that have brain-specific expression pattern account for ∼19 % of the total (tissue-specific genes were identified using the TSEA tool, http://doughertytools.wustl.edu/TSEAtool.html). Panel C – genes involved in development or growth or morphogenesis account for ∼29 % of the total. Panel D – genes encoding receptors, whose ligands are of a peptide nature, account for ∼56 % of the total.

We found that more than 80 % of the total number of genes (67 of 80) encoded G protein-coupled receptors (Figure 1A). The genes belonging to the GPCR superfamily are marked with the number 1 in the fourth column of Table S2.

Using TSEA tool (see Section 2.3), we identified subsets of genes that had tissue-specific expression patterns in a number of tissues or organs. The maximum number of genes with tissue-specific expression pattern (15 genes, or 18.75 %) were specific to the brain (Figure 1B). It was also found that the brain-specific genes were overrepresented (*p* < 0.05) in the *Receptors_80* set (Table S3). The genes that had brain-specific expression pattern are marked with the number 1 in the fifth column of Table S2.

Additionally, we identified genes within the *Receptors_80* set involved in the regulation of developmental processes. Using DAVID, we found genes associated with GO terms containing the words *development*, *growth* and *morphogenesis*. This analysis revealed that 28.75 % of genes (or 23 out of 80) from the *Receptors_80* set may be involved in developmental processes (Figure 1C). The GO term annotations for these 23 genes from the GOTERM_BP_DIRECT dictionary containing words *development*, *growth* and *morphogenesis* are provided in Table S4. Genes associated with developmental processes are marked with the number 1 in the sixth column of Table S2.

We found published evidence supporting the participation of these 23 genes in the regulation of developmental processes (presented in Table S5). Several genes from this set (*EPHA3, BMPR1A, LGR4, TLR4, AGTR2, NPY1R, NPY5R,* etc.) were found to be involved in the regulation of development at the embryonic stage [20], [56], [57], [58], [59], [60], [61], [62], [63], [64], [65]. The *DRD1, LEPR, NTRK2, NPY1R, NPY2R, OPRD1*, and *CRHR1* had evidence of their participation in the control of growth, differentiation, or morphogenesis of neuronal cells [17], 21], [66], [67], [68], [69], [70], [71], these findings are highlighted in red in the third and the fourth columns of Table S5. Additionally a number of genes (*BMPR1A, LGR4, NTRK2*) have been implicated in the control of jaws and teeth formation and development [59], [72], [73], [74], [75], these findings are highlighted in blue in the third and the fourth columns of Table S5.

We have classified genes from the *Receptors_80* set according to the types of ligands to which the receptors encoded by these genes may bind. We found that the receptors encoded by the genes from the *Receptors_80* set interact with 8 types of ligands (see the seventh column of Table S2). Over half of the genes (45 genes, or ∼56 % percent) encode receptors that interact with ligands of peptide nature (Figure 1D). Eleven receptors interact with protein ligands, eight receptors interact with lipid ligands, seven receptors interact with aminergic ligands, three receptors interact with ligands of two other types and eight of the 80 receptors were classified as orphan.

It was found that four genes belonging to the GPCR superfamily are both brain-specific and related to the regulation of developmental processes. These genes (*CCKBR, NPY2R, DRD1*, and *DRD2*) are marked with the number 1 in the fourth, fifth and sixth columns of Table S2. Receptors encoded by two of these genes (*CCKBR* and *NPY2R*) are activated by peptide ligands (gastrin and cholecystokinin for CCKBR and neuropeptide Y for NPY2R), while the other two receptors (DRD1 and DRD2) bind aminergic ligand (dopamine).

### 3.3 Analysis of the evolutionary characteristics of genes encoding cell surface receptors and involved in appetite regulation

#### 3.3.1 The analysis scheme

**Figure 2:**
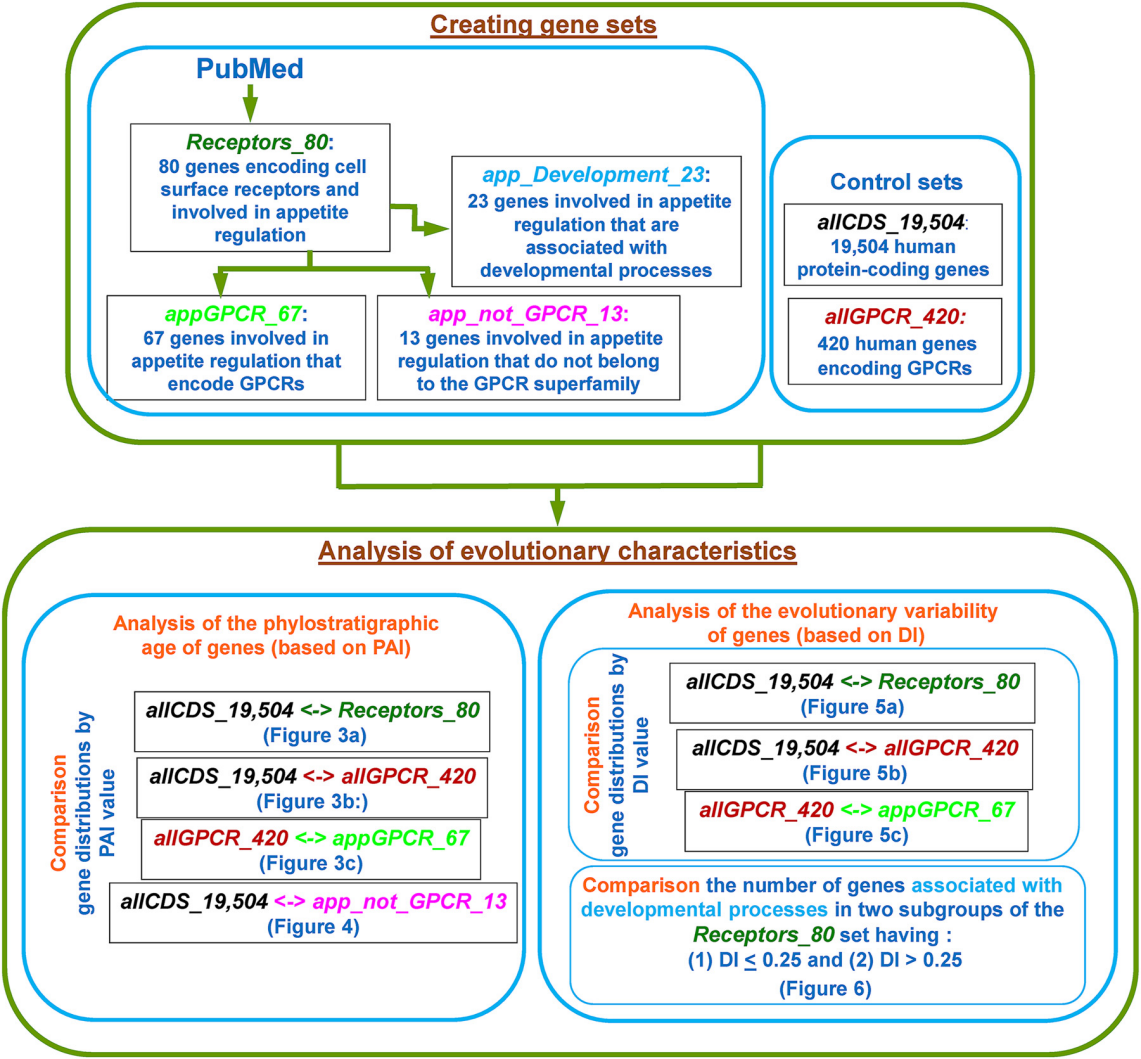
The general scheme of the analysis of the evolutionary characteristics of human genes encoding cell surface receptors and regulating appetite. A description of the gene sets is presented in Table 1 as well as in Sections 2.1 and 2.2. The PAI (phylostratigraphic age index) and DI (divergence index) are the evolutionary characteristics of genes, characterizing evolutionary age of genes and the type of selection to which they were subjected.

The general scheme of the analysis is shown in Figure 2. Based on the *Receptors_80* set we formed three subsets: (1) *appGPCR_67* – genes from the *Receptors_80* set that encoded GPCRs; (2) *app_not_GPCR_13* – genes from the *Receptors_80* set encoding receptors that do not belong to the G protein-coupled receptor superfamily; (3) *app_Development_23* – genes from the *Receptors_80* set that may be involved in developmental processes.

Determining the phylostratigraphic age index of genes relies primarily on comparing amino acid sequences of encoded proteins [76], 77]. The DI index is calculated from DNA sequences located in exons. Therefore, in order to determine how unique the evolutionary features of receptor genes involved in appetite regulation are in comparison with other human genes, we formed gene set comprising human protein-coding genes (*allCDS_19,504*). The set *allCDS_19,504*, includes 19,504 human protein-coding genes, for which PAI and DI values could be calculated (see Section 2.6).

Since most of the genes from *the Receptors_80* set (67 out of 80) encoded receptors from the GPCR superfamily, it seemed useful to investigate the evolutionary characteristics of this subgroup by comparing them with all the genes encoding GPCRs. Therefore, we formed the set *allGPCR_420*, which contained human genes encoding G protein coupled receptors (this set included genes from the GPCRdb database (https://gpcrdb.org)). Additional information about these sets is provided in Table 1.

Next, we investigated the evolutionary characteristics of appetite-regulating genes. We used PAI (phylostratigraphic age index), which characterizes the evolutionary age of the gene, and the DI (divergence index), indicating the type of selection affecting the gene (see Section 2.6). In the course of analysis, we constructed distributions of PAI and DI values of genes, and compared them with the similar distributions obtained for all human protein-coding genes (*allCDS_19,504*), as well as for genes encoding GPCR superfamily receptors (*allGPCR_420*). We also analyzed the content of genes involved in developmental processes in two subsets of appetite-controlling genes, categorized by their DI values (the first subset – DI less than or equal to 0.25, and the second subset contained other genes, that is, genes with DI more than 0.25).

#### 3.3.2 Evaluation of the phylostratigraphic age of genes encoding cell surface receptors involved in appetite regulation (PAI-based analysis)

To estimate the phylostratigraphic age, the phylostratigraphic age index (PAI) was used, which takes a value from 1 to 16. PAI values indicate evolutionary stages corresponding to the stages of divergence of certain taxa, as it is described in Section 2.6.

##### 3.3.2.1 Comparison of *Receptors_80* and *allCDS_19,504* sets

**Figure 3:**
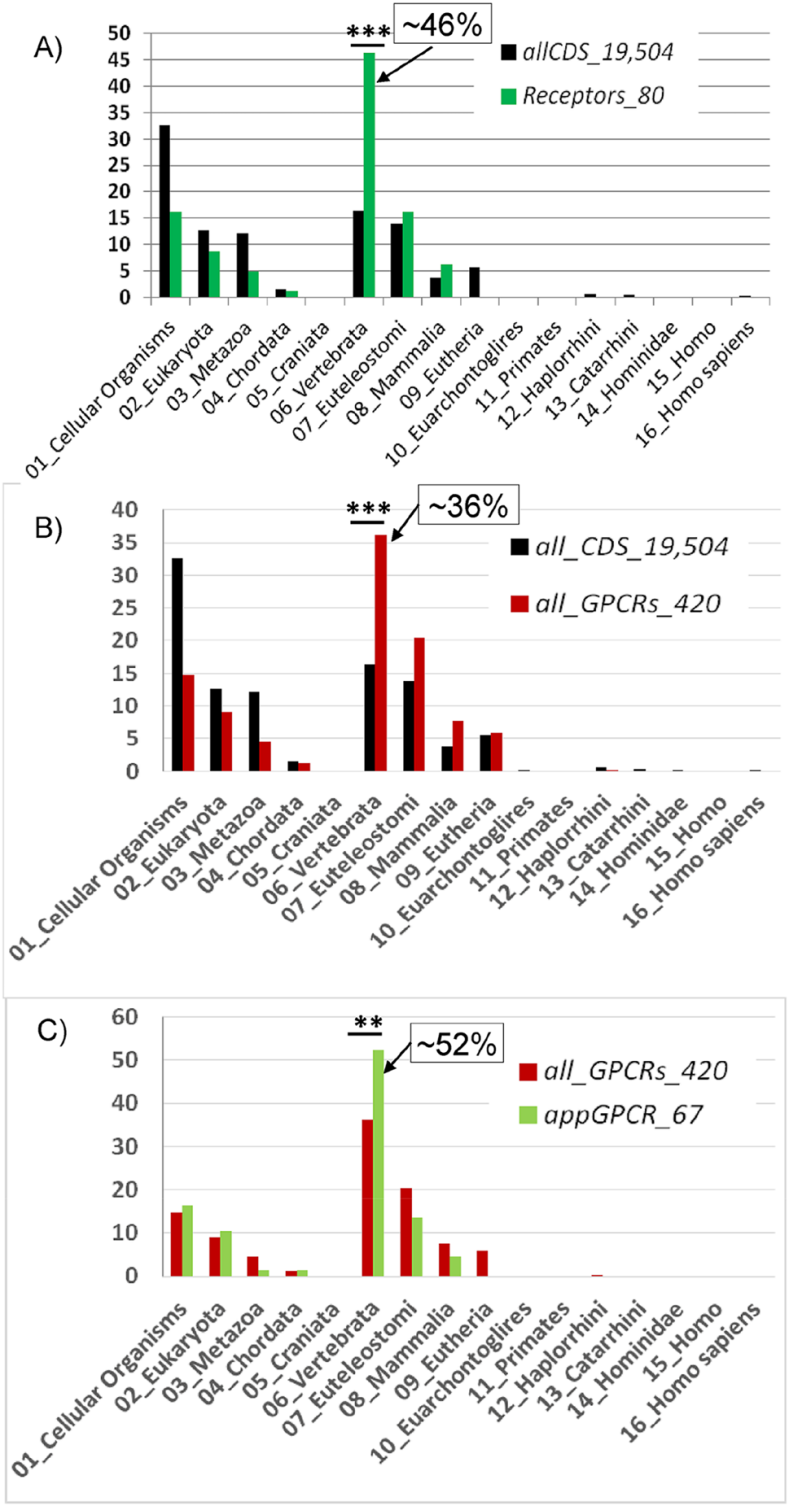
The distributions of the sets of human genes presented in Table 1 according to PAI values. In all graphs, the X-axis corresponds to PAI, indicating the evolutionary stages of taxon divergence (approximate dates (in in millions of years ago) corresponding to the stages of divergence of these taxa are shown in Table 2). In all graphs, the Y-axis shows the percentage of genes. (A) The comparison of all human protein-coding genes, *allCDS_19,504* (control set), and the human appetite-regulating genes encoding receptors, *Receptors_80* set; (B) the comparison of all human protein-coding genes, *allCDS_19,504* (control set), and genes encoding all GPCRs, *allGPCR_420* set; (C) the comparison of genes encoding all human GPCRs, *allGPCR_420* (control set), and genes encoding GPCRs controlling appetite, *appGPCR_67* set. PAI values were calculated by the Orthoscape software tool at a threshold of 0.5 for the level of similarity between the DNA sequences of the orthologous genes (see Section 2.6). Asterisks indicate differences between the number of genes with a PAI equal to 6 (the stage of *Vertebrata* divergence) and their expected numbers calculated based on the distributions in the control sets. ***p < 0.001, **p < 0.01. See Tables S7, S8 and S9.

Using phylostratigraphic age index (PAI) we compared phylostratigraphic age of human genes from the *Receptors_80* set (Table S6) with the same characteristic defined for all human protein-coding genes (the *allCDS_19,504* set) (Figure 3A). The distributions of genes from the both sets according to PAI values were uneven. A substantial part of protein-coding genes from the *allCDS_19,504* set (∼33 %, 6,350 genes) had PAI equal to 1 (Cellular organisms, the root of the phylogenetic tree) and about 16 % and 14 % of genes (3,203 genes and 2,708 genes, respectively) had PAI equal to 6 (the stage of Vertebrata divergence) and 7 (the stage of Euteleostomi divergence), respectively. When considering the same distribution for 80 genes encoding cell surface receptors and involved in appetite regulation (the set *Receptors_80*), the number of genes with PAI equal to 6 (the stage of Vertebrata divergence) was the most abundant (37 out of 80 genes which equals ∼ 46 %). This number (37) was significantly (*p* < 0.001) higher than the expected number (13.14) calculated based on the distribution obtained for the gene set *all_CDS_19,504* (Figure 3A, Table S7). Thus, according to the results of our analysis (Figure 3A), the ancestral forms of a significant part (∼46 %) of the genes encoding receptors and regulating appetite (*Receptors_80* set) formed at the stage of Vertebrata divergence. Notably, this period coincides with the formation of teeth and jaws in vertebrates, enabling new strategies of eating behavior formed (from finding food to its absorption and digestion).

##### 3.3.2.2 Comparison of *allGPCR_420* and *allCDS_19,504* sets

As noted earlier, most genes from the *Receptors_80* set (67 genes out of 80) encode GPCRs (Figure 1A). Therefore, this and the subsequent steps of the analysis were performed to determine to what extent the evolutionary features of the genes from the *Receptors_80* set are caused by the features of genes from the GPCR superfamily. We analyzed the distribution of PAI values for a set of 420 human genes encoding GPCRs (*allGPCR_420* set compiled from GPCRdb data, https://gpcrdb.org). We compared this distribution with a similar characteristic defined for all genes encoding human proteins (*allCDS_19,504* set). It turned out that both distributions were non-uniform (Figure 3B). The number of genes in the set *allGPCR_420* that had PAI values equal to 6 (the stage of Vertebrata divergence) was 152 (∼36 %) significantly exceeding the expected 68.97 (*p* < 0.001; Table S8). Thus, firstly, a significant part of the ancestral forms of genes encoding GPCRs were formed at the stage of Vertebrata divergence (Figure 3B), that is, at the same evolutionary stage as most of the genes from the genome-wide sample. Secondly, the *allGPCR_420* set containing all genes encoding GPCRs has its own evolutionary features that distinguish it from a genome-wide set composed of all human protein-coding genes (*allCDS_19,504*).

##### 3.3.2.3 Comparison of *appGPCR_67* and *allGPCR_420* sets

The distinctive features of the PAI distributions for the genes from the *allGPCR_420* set (Figure 3B) and the *Receptors_80* set (Figure 3A) are similar: both differ comparably from the distribution obtained for a genome-wide *allCDS_19,504* set. Therefore, the purpose of the next step of our analysis is to determine whether PAI distribution for *Receptors_80* set reflects the evolutionary characteristics of the entire GPCR superfamily (*allGPCR_420* set) or this set has its own specific evolutionary features. To address this we compared the distribution of PAI values for the following two sets of genes: (1) *appGPCR_67* (appetite-regulating GPCR-encoding genes); (2) *allGPCR_420* (all GPCR-encoding genes from GPCRdb) (Figure 3C). Within *appGPCR_67* set, 35 genes (52.2 %) were found to have a PAI equal to 6 (the stage of Vertebrata divergence), which was significantly (*p* < 0.01) higher than the expected value of 24.25 calculated based on the proportion of this group of genes in the set *allGPCR_420* (Table S9). Thus appetite-regulating GPCRs (*appGPCR_67* set) possess specific evolutionary features distinguishing them from the broader GPCR superfamily (*allGPCR_420*).

##### 3.3.2.4 Comparison of *app_not_GPCR_13* and *allCDS_19,504* sets

**Figure 4:**
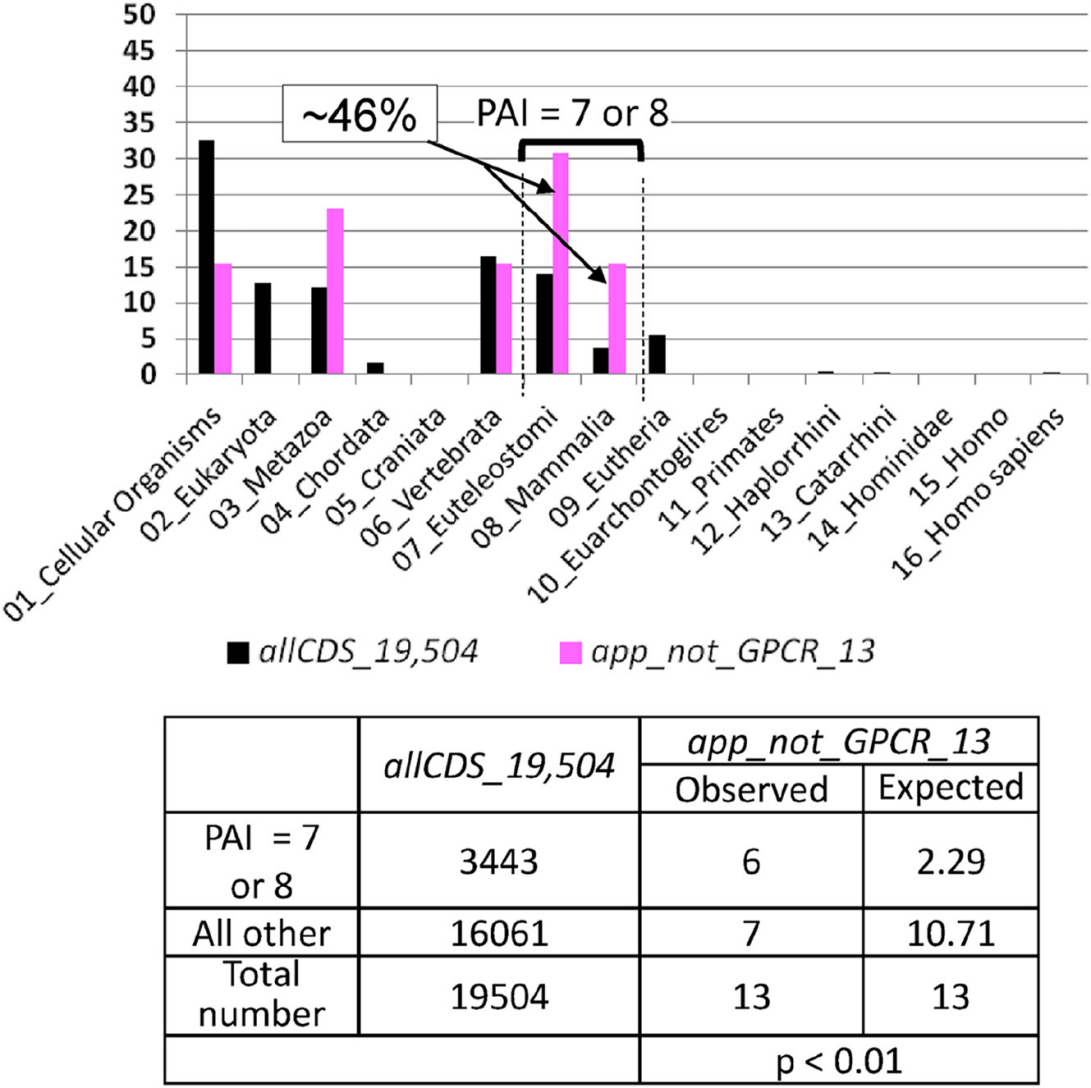
The distributions of PAI values for all human protein-coding genes, *allCDS_19,504* (control set), and genes controlling appetite but not belonging to GPCR superfamily, *app_not_GPCR_13* set. The X-axis corresponds to PAI (see Table 2), the Y-axis shows the percentage of genes. PAI values were calculated by the Orthoscape software tool at a threshold of 0.5 for the level of similarity between the DNA sequences of the orthologous genes (see Section 2.6).

It was previously stated that 13 genes from the *Receptors_80* set did not belong to the superfamily of G protein-coupled receptors (these genes were included in the *app_not_GPCR_13* gene set). We aimed to identify the specific evolutionary features of this group of genes. We found that the distribution of PAI values for this set of genes differs from that of all human protein-coding genes (Figure 4). In this case, a significantly higher number of genes than expected (*p* < 0.01) were found in the group of genes with PAI equal to 7 or 8 (the stages of Euteleostomi and Mammalia divergence). In the *app_not_GPCR_13* set, six out of 13 genes had PAI values of 7 or 8 (46.1 %), and in the *allCDS_19,504* set, this PAI range was observed in 3,443 genes (17.7 %). This indicates expected number of genes with these PAI values in the *app_not_GPCR_13* set must be 2.29 (see the table at the bottom of Figure 4). Thus, although the genes of the *app_not_GPCR_13* set do not belong to the GPCR superfamily (Table 1), they exhibit specific evolutionary characteristics: ancestral forms of these genes originated during evolutionary stages corresponding to the stages of Euteleostomi and Mammalia divergence significantly more frequently than the ancestral forms of the entire set of protein-coding genes (*allCDS_19,504*).

Thus, according to the results of our analysis, a significant proportion of the genes encoding receptors involved in regulation of appetite (as well as genes from the subset belonging to the GPCR superfamily) has a phylostratigraphic age index corresponding to the stage of Vertebrata divergence. At the same time, the set of non-GPCR appetite receptor genes shows an elevated proportion (46 %) of genes having phylostratigraphic age corresponding to the stages of Euteleostomi and Mammalia divergence.

#### 3.3.3 Evolutionary variability of genes (DI-based analysis)

To analyze the evolutionary variability, we used the divergence index (DI) (see Section 2.6). DI indicates the type of selection to which the gene under study is subjected. DI value below 1 indicates that the gene is subjected to stabilizing selection, while DI value above 1 indicates that the gene is subjected to driving selection.

##### 3.3.3.1 Comparison of *Receptors_80* and *allCDS_19,504* sets

**Figure 5:**
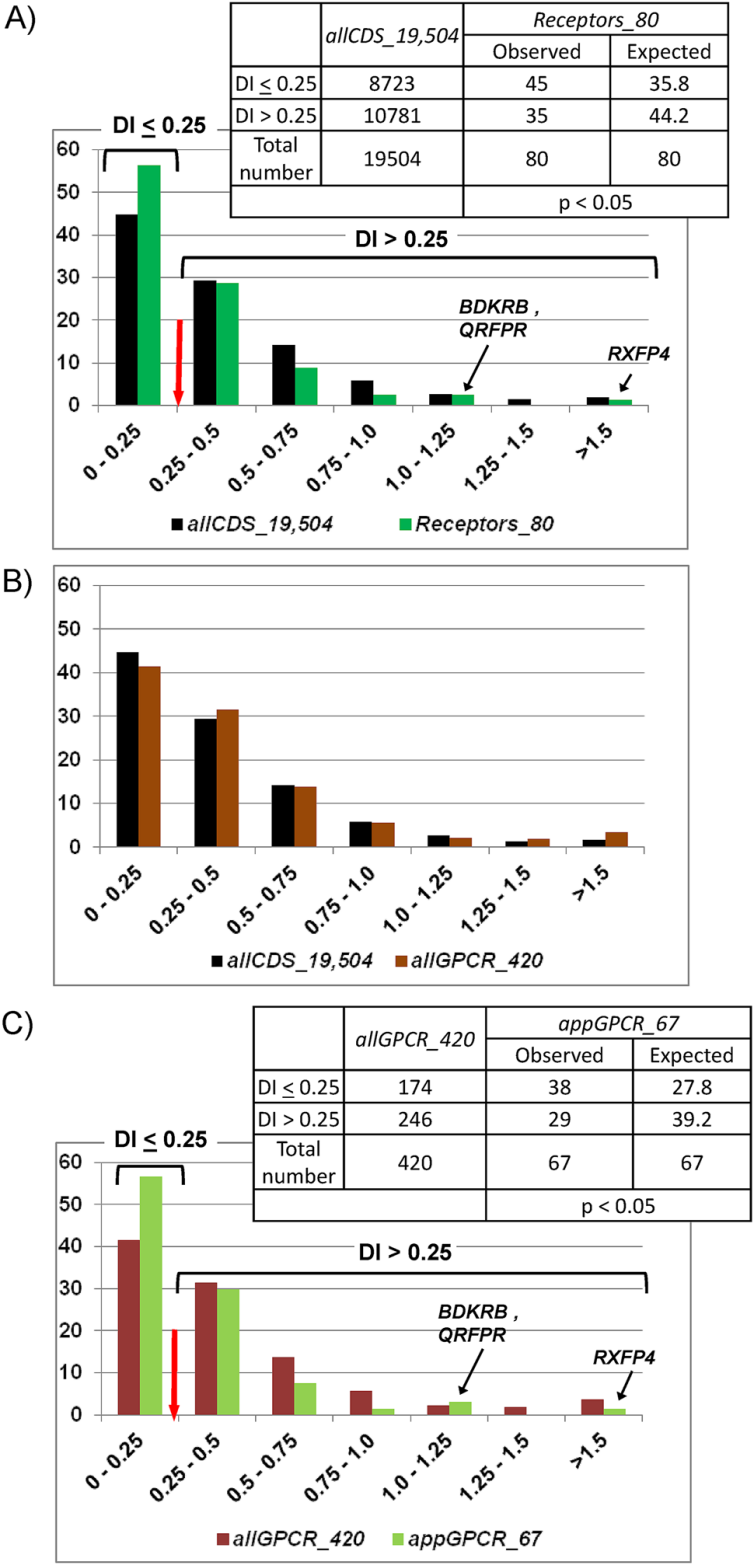
The distributions of genes from the sets presented in Table 1 according to the DI values. In all graphs, the X-axis corresponds to the values of DI, and the Y-axis indicates the proportion of genes (as a percentage) with a certain DI value. (A) The comparison of all human protein-coding genes, *allCDS_19,504* (control set), and the human appetite-regulating genes encoding receptors, *Receptors_80* set. The observed and expected number of genes with DI ≤ 0.25 and DI > 0.25 are presented in the table above the graph; (B) the comparison of all human protein-coding genes, *allCDS_19,504* (control set), and genes encoding all GPCRs, *allGPCR_420* set. (C) The comparison of genes encoding all human GPCRs, *allGPCR_420* (control set), and genes encoding GPCRs controlling appetite, *appGPCR_67* set. The observed and expected numbers of genes having DI ≤ 0.25 and DI > 0.25 are presented in the table above the graph.

We generated distributions of the human protein-coding genes (*allCDS_19,504*) and genes from the *Receptors_80* set according to DI values (Figure 5A). The three genes with the highest DI values (1.84, 1.21 and 1.03) from the *Receptors_80 set* were: *RXFP4, QRFPR*, and *BDKRB1* (the DI values for the genes from the *Receptors_80* set in ascending order are given in Table S10). The *Receptors_80* set contained a small proportion of genes (3.75 %) that had DI value more than 1, while in the *allCDS_19,504* set the proportion of genes with DI value more than 1 was 5.85 %. It has been found that 44.7 % of all protein-coding genes exhibited DI less than or equal to 0.25. For the *Receptors_80* set, the proportion of genes with low DI (below or equal to 0.25) was 56.3 % (45 out of 80) and it was higher (*p* < 0.05) than the expected number (35.8) calculated based on the distribution obtained for the *allCDS_19,504* set (Figure 5A). So, the *Receptors_80* set contained an increased proportion of genes with DI values below 0.25. This indicates that genes from the *Receptors_80* set undergo stabilizing selection to a greater extent than genes from the set of all human protein-coding genes as a whole.

##### 3.3.3.2 Comparison of *allGPCR_420* and *allCDS_19,504* set

We detected no significant difference between the distributions over DI values obtained for the set of all receptors from the GPCR superfamily (*allGPCR_420*) and the *allCDS_19,504* set (Figure 5B).

##### 3.3.3.3 Comparison of *appGPCR_67* and *allGPCR_420* sets

We compared the distribution of genes from the *appGPCR_67* set (encoding appetite-regulating GPCRs) over DI values with the distribution of all receptors from the GPCR superfamily (*allGPCR_420*) (Figure 5C). The number of genes with low DI (DI ≤ 0.25) in the *appGPCR_67* set (38 genes) was significantly (*p* < 0.05) higher than the expected number of genes (27.8) calculated from the DI distribution for all genes encoding GPCRs (Figure 5C, table above the graph). This indicates that genes encoding GPCRs controlling appetite (*appGPCR_67* set) undergo stabilizing selection to a greater extent than all human genes encoding GPCR (*allGPCR_420* set).

##### 3.3.3.4 Analysis of the genes of the *Receptors_80* set by the DI value and their association with developmental processes

**Figure 6:**
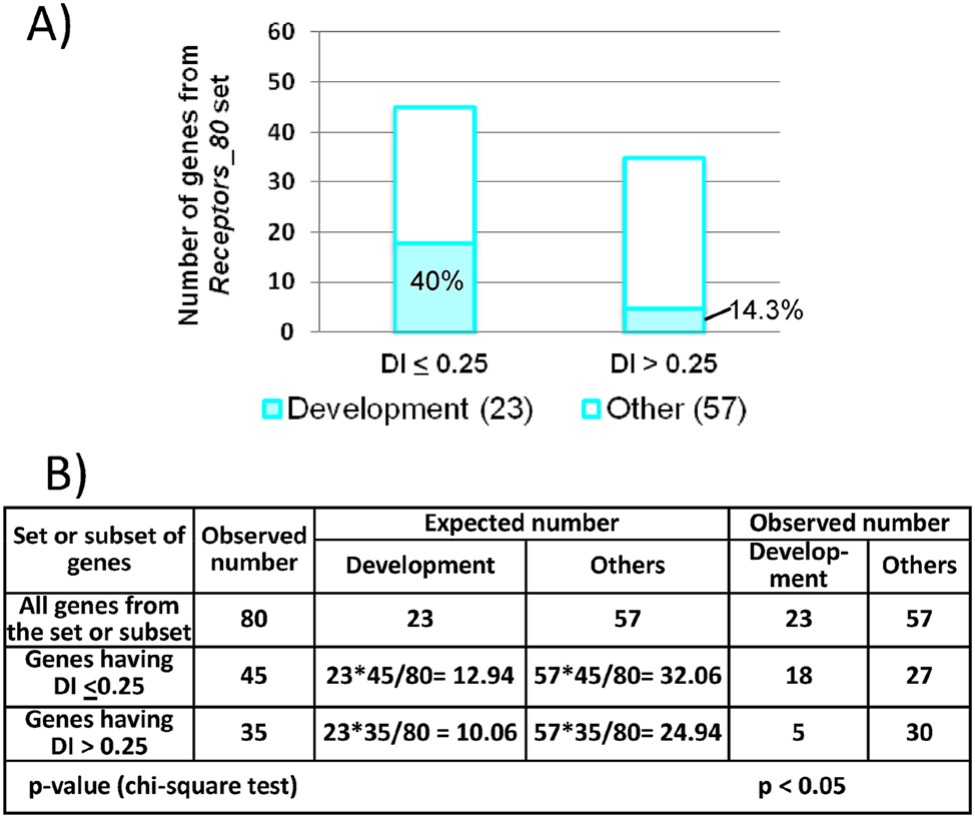
Distribution of genes from the Receptors_80 set by DI values. (A) The proportions of genes that according to DAVID are associated with developmental processes are shown. (B) According to the chi-square criterion, the observed numbers of genes associated with developmental processes significantly (*p* < 0.05) differed from the expected number.

Earlier, we found that the *Receptors_80* set is enriched with genes exhibiting DI values below or equal to 0.25 (Figure 5A). Therefore, we next analyzed the functions of appetite-regulating genes within this low-DI subgroup (DI ≤ 0.25) focusing on their involvement in the regulation of developmental processes. As indicated above, approximately one third (28.75 %) of genes from the *Receptors_80* set are involved in development, growth or morphogenesis. For brevity, we designated this group as the *app_Development_23* set (these genes are marked with the number 1 in the sixth column of Table S2 and are listed in Table S4). The *Receptors_80* set was divided into two subgroups according to the value of the DI index: (1) a subgroup of genes with low DI (DI ≤ 0.25); (2) a subgroup including all other genes (their DI values ranged from 0.25 to 1.84). Within each subgroup, we determined the number of genes associated with developmental processes (i.e., belonging to the *app_Development_23* set). We found that 40 % of genes in the low-DI subgroup (DI ≤ 0.25) were associated with development, compared to only 14.3 % in the subgroup with DI > 0.25 (Figure 6A). The number of genes associated with development in these two subsets differed significantly (*p* < 0.05) from random expectation (Figure 6B). In the subgroup of genes with low DI (DI ≤ 0.25), the observed number was 18 genes (40 %), while the expected number was 12.94. In the subset of genes with DI > 0.25, 5 genes (14.3 %) were detected, and the expected value was 10.06 (Figure 6B). Thus, the subgroup of genes with low DI (≤0.25) is enriched with genes associated with development, while the subgroup of genes with DI > 0.25 shows depletion.

## 4 Discussion

### 4.1 Genes regulating appetite and functional characteristics of encoded proteins

In this work, we classified human genes encoding cell surface receptors involved in appetite regulation according to the structure of receptors, involvement in developmental processes, tissue-specific characteristics, the evolutionary stage when the ancient form of gene emerged, and evolutionary variability.

Cell surface receptors are transmembrane proteins that play a very important role in the cell. These proteins interact with various molecules located in the extracellular space (ligands) and activate signal transduction pathways in the cell [28], 78]. Genes encoding cell surface receptors constitute a substantial portion of the human genome (more than a thousand genes) [28]. The activity of cell surface receptors may be modulated by a wide variety of biochemical compounds including pharmaceuticals that are called agonists and antagonists [28].

Here we present a set comprising 80 human genes encoding cell surface receptors, whose orthologs regulate food intake in model organisms. This set includes 16 more genes than in the previous study [29]. These 16 additional genes are *ADCYAP1R1, BDKRB1, CALCR, CMKLR1, CNR1, EPHA3, ESR1, FFAR3, GPBAR1, GPR39, GPR75, HTR2C, KISS1R, LRP1, OR51E2, RXFP4* (Figure S1). The discovery of so many genes controlling eating behavior supports the idea that the regulation of food intake is a very complex process. This regulation is carried out through the coordinated networks, including both gene networks in various parts of the brain and gene networks in peripheral organs and tissues. Additionally, appetite can fulfill the body’s basal need for nutrients (homeostatic appetite, fulfilling physiological needs for energy) and the need for new experiences associated with food (non-homeostatic appetite that satisfies the need for positive emotions) [9], 79], 80]. These two forms of feeding behavior are controlled by distinct neuronal populations localized in distinct brain areas [9]. It is known that food motivation depends (1) on signals received from the sensory organs and (2) on information about the state of various physiological systems of the body [6], [81], [82], [83]. Processing of this information is performed by specialized nerve cells that express a wide range of receptors [3], 8]. In this way, the desire to consume food can be adjusted depending on psycho-emotional state of an individual or the life situation (chronic stress, depression, fright, boredom, for animals – mating behavior, territory protection, threat from predators, etc.) [84], [85], [86], [87], [88]. The intensity of food motivation may also depend on (1) the rhythm of sleep and wakefulness (circadian rhythm) [89]; (2) signals coming from the placenta and the developing fetus during pregnancy [90], 91]; (3) the onset of lactation [90]; (4) the phase of menstrual cycle, which occurs in female individuals [92].

### 4.2 Low evolutionary variability of genes encoding receptors involved in appetite regulation

Analysis of the distribution of DI values (Table S10) revealed a significant enrichment of the *Receptors_80* gene set with genes undergoing stabilizing selection (Figure 5A). The set of appetite-regulating genes encoding GPCRs (*appGPCR_67*) similarly showed increased low-DI gene content (Figure 5C). Among all the genes from the *Receptors_80* set, 11 genes with very low of DI values (DI < 0.05) were found: *GPR26, NPY1R, GHSR, CNR1, ADIPOR1, DRD1, MCHR1, ADCYAP1R1, NPY2R, GPR171, NPBWR1* (Table S10). These values suggest that these genes were subjected to strong pressure of stabilizing selection. The characteristics of these eleven genes are presented in Table S11. Ten of these 11 genes (with the exception of *ADIPOR1*) encode GPCRs.

*GPR26* showed the lowest DI (DI < 0.005). This gene encodes GP26 receptor, an orphan receptor from the GPCRs superfamily. This orphan GPCR’s inactivation in mice causes hyperphagia [93]. It had also been shown that mice with GP26 receptor deficiency are prone to depression, characterized by increased anxiety, as well as increased cravings for ethanol [94]. TSEA tool [34] has indicated that human *GPR26* has brain-specific expression pattern. Its expression is found in areas of the human brain such as the amygdala, hippocampus, and thalamus [95]. The mechanism of GP26 receptor action is poorly understood. It is known that GPR26, like a number of other receptors (5-HT1B, 5-HT1D, EDG1, EDG3, and GABAB2 receptors), is able to form heterodimers with the 5-hydroxytryptamine receptor 1A (HTR1A) [96], thus suggesting potential chaperone activity, stabilizing other receptors from GPCRs superfamily, as does σ1 receptor, encoded by *SIGMAR1* [97], 98]. *GPR26 c*onservation is further supported by functional homology: the genome of a fairly distant model species (*Caenorhabditis elegans*) contains *Y5H2B* gene, which is similar to the human *GPR26*. And it turned out that *Y5H2B* is involved in the regulation of fat content [99].

A remarkable feature of the group of genes with DI value less than 0.05 is that most of them (8 out of 11) have a phylostratigraphic age PAI = 6, corresponding to the stage of Vertebrata divergence. The probability of observing such a grouping of genes occurring randomly is less than 1*10^−6^. Increasing the threshold of the DI value to 0.1, identified 21 genes in the *Receptors_80* set and 14 of these 21 genes having PAI = 6. The corresponding *p*-value in this case is less than 1*10^−9^. Thus, this assessment is stable. The reason that most of the genes from the group under consideration (Table S11) have a high level of conservatism and the same PAI = 6 (that is, they formed in a coordinated manner) may be related to some essential Vertebrata adaptations associated with ancestral gene formation.

The analysis also revealed two genes, *RXFP4* and *QRFPR* (DI values were 1.84, and 1.21, respectively), under driving selection [30]. The characteristics of these genes are also shown in Table S11.

*RXFP4* encodes relaxin family peptide/INSL5 receptor 4 that binds relaxin 3 and insulin-like peptide 5 (Insl5). Relaxin 3 and Insl5 belong to the insulin superfamily and play a role in appetite regulation. Insl5 is an orexigenic hormone released from colonic L-cells. Intraperitoneal injection of Insl5 increased food intake in wild-type mice but not mice lacking the cognate receptor Rxfp4 [100].

*QRFPR* (pyroglutamylated RFamide peptide receptor) encodes a receptor for a neuropeptide from the RFamide family. It was shown that in mice central administration of pyroglutamylated arginine-phenylamine-amide peptide (QRFP/26RFa) increased both food intake and locomotor activity [101].

The two genes considered (*RXFP4* and *QRFPR* both having DI > 1) are relatively “young”, they have the same phylostratigraphic age: their PAI value is 7 (the stage of Euteleostomi divergence). Therefore, these genes could evolve adaptively, because they had no time to be engaged in a large number of interactions in the gene networks regulating biological processes, facilitating driving selection.

### 4.3 Low evolutionary variability of genes encoding receptors involved in appetite regulation and associated with developmental processes

Approximately 29 % of receptors in *Receptors_80* set were involved in developmental processes (Figure 1C, Table S5). The data from Table S5 demonstrate that the receptor genes involved in appetite regulation can participate in the control of a wide range of developmental processes occurring both at the prenatal and postnatal stages of ontogenesis. This fact indicates the existence of an additional mechanism linking the processes of growth and development with the control of food intake.

Notably, several *Receptors_80* genes participate in developmental pathways seemingly unrelated to appetite regulation. Many genes are pleiotropic, that is, they are involved in the regulation of completely different biological processes. For example, alpha-melanocyte stimulating hormone (α-MSH) and its receptor, MC1R, are known as a master regulators of melanogenesis. At the same time, their pleiotropic effects beyond pigmentation are discovered [102], including antibacterial and antifungal activity [103]. Another example is oxytocin and its receptor (OXTR). In addition to its uterine-contracting activities and the ability to stimulate lactation [104], oxytocin receptor influences regulation of many processes. Mice deficient in the Oxtr gene (Oxtr−/−) displayed dysfunction in body temperature control when exposed to cold, aberrations in social behaviors (male aggression, mother-offspring interaction), and were prone to obesity susceptibility [105].

Analyzing the evolutionary variability of genes encoding receptors from *Receptors_80* set (DI-based analysis), we found that the subgroup of genes with low DI (less than or equal to 0.25) is enriched with genes associated with developmental processes, while the subgroup of genes with higher DI (more than >0.25) is characterized by reduced content of genes associated with developmental processes (Figure 6). Forty percent of the genes (that is, 18 out of 45) having DI less than or equal to 0.25 were classified as genes associated with development. The proportion of such genes among genes with DI less than 0.05 was ∼55 % (six out of eleven) (Table S11). These six genes are: *NPY1R, GHSR, CNR1, ADIPOR1, DRD1*, and *NPY2R* (listed in ascending order of DI). Evidence of the involvement of these genes in the regulation of developmental processes is shown in Table S5. The functions of these genes are briefly described below.

*NPY1R* and *NPY2R* encode receptors mediating the action of neuropeptide Y (NPY), a neurotransmitter, and peptide YY (PYY), a gastrointestinal hormone. NPY1 and NPY2 receptors mediate neuroprotective and neurogenic effects of centrally administered NPY in Alzheimer’s mouse models [21].

*GHSR* encodes a receptor for ghrelin, an orexigenic hormone secreted by endocrine cells of the stomach and duodenum [106], 107]. Ghrelin stimulates growth hormone release in humans [19].

*CNR1* encodes one of two receptors for cannabioids. CNR1 deficiency in dorsal telencephalic glutamatergic neurons prevented the overconsumption of palatable food in knockout mice [52]. Pharmacologic blockade of peripheral *Cnr1* signaling inhibits pituitary growth hormone pulsatile secretion in mice [108], suggesting developmental regulation.

*ADIPOR1* encodes a receptor for adiponectin, a hormone secreted by adipocytes. Studies in rats have shown that ADIPOR1 mediates the anorexigenic and insulin/leptin-like actions of adiponectin in the hypothalamus [109]. It was also shown that in a HEK293 cell model adiponectin receptor AdipoR1 activate ERK1/2 through a Src/Ras-dependent pathway and stimulate cell growth [110].

*DRD1* encodes a D1 subtype of the dopamine receptor. It is the most abundant dopamine receptor in the central nervous system, and it plays an important role in the development of human nervous system promoting differentiation of the neural stem cells [66]. The effect of selective D1 dopamine receptor agonists on food consumption were investigated in free-feeding rats. A selective D1 receptor agonist decreased food pellet intake [111].

These development-associated appetite genes exhibit extremely low DI values (<0.05), indicating strong stabilizing selection. This fact is consistent with the idea that food intake is closely related to developmental processes, including cell differentiation, growth, and morphogenesis.

### 4.4 Important events in the evolution of the development and regulation of appetite (phylostratigraphic age of genes)

#### 4.4.1 Phylostratigraphic age of genes

Phylostratigraphy, a method for estimating the evolutionary age (phylostratum) of genes based on inferred presence or absence of their homologs across a phylogenetic tree, relies on several assumptions.

The fundamental prerequisite of phylostratigraphy is the accurate inference of homology relationships as well as the correct classification of genes as orthologs (resulting from speciation events) versus paralogs (resulting from gene duplication events [112]. Phylostratigraphy traces a gene’s origin back to the node representing the last common ancestor (LCA) where an ortholog of that gene is inferred to have been present. Misclassification, such as mistaking an in-paralog (arising after a speciation event) for an ortholog, or failing to detect an out-paralog (arising before a speciation event) may fundamentally distort this tracing, potentially making a gene appear older (if paralogs are mistaken for orthologs) or younger (if true orthologs are missed or misclassified as recent paralogs) than it actually is [77].

In the context of phylostratigraphic analysis, following their origin, genes are assumed to evolve primarily through a process of gradual, phyletic evolution. This implies that their sequence changes incrementally over time, preserving sufficient similarity to be detectable by standard homology search methods (e.g. BLAST, HMMER) throughout descendant lineages, barring explicit gene loss events [113]. If a gene undergoes rapid, radical sequence divergence shortly after its origin, or experiences strong diversifying selection leading to a major functional shift, its sequence similarity to its ancestral state or orthologs in other lineages may fall below detectable levels. Several studies highlight how rapid evolution can lead to homology detection errors and thus bias phylostratigraphic age estimates [114], [115], [116].

The phylogenetic tree used must provide sufficient taxonomic sampling across the lineages of interest to accurately capture the true pattern of homology presence and absence. Phylostratum boundaries are defined by the distribution of gene presence across tree nodes. Sparse sampling may create gaps in this distribution [77], 113].

As noted above, a significant proportion of genes encoding receptors involved in appetite regulation is associated with development (see Table S2, Figure 1C). Therefore, in the further discussion, we focus on the role of these genes in developmental processes in the context of significant events in the evolution of embryogenesis and morphogenesis, as well as taking into account the phylostratigraphic age of these genes (based on the PAI value).

#### 4.4.2 Ancient genes for appetite regulation and development (PAI = 1 and 2)

These evolutionary stages correspond to the emergence of unicellular eukaryotic organisms. Analysis of PAI values (Table S6) showed that among the genes involved in appetite regulation, there are so-called “ancient” ones, that is, having PAI = 1 (root of the taxonomic tree), or PAI = 2 (the stage of Eukaryota divergence) (Figure 3A). Ancestral forms of these genes appeared at the stage of unicellular eukaryotic organisms. During subsequent evolutionary specialization genes of this group acquired a number of specific functions. For example, in mammals: (1) CCKBR (cholecystokinin B receptor), the receptor for cholecystokinin, a gastrointestinal satiety signal released from the duodenum and terminating feeding [117]; (2) CCKAR (cholecystokinin A receptor), a paralog of the CCKBR with the same function; (3) GLP2R (glucagon like peptide 2 receptor), the receptor for glucagon-like peptide-2 which can be secreted in the brain and reduce food intake [118]; (4) INSR, the receptor for insulin which, along with other effects, can control production of alpha-melanocyte-stimulating hormone and neuropeptide Y by neurons located in the arcuate nucleus [119]; (5) NTRK2, the receptor for the brain derived neurotrophic factor (BDNF), a potent regulator of neuronal activity and neurogenesis [120], which is known to cause anorexic effect [18]. According to data available from the Expression Atlas (https://www.ebi.ac.uk/gxa/home), these genes of ancient origin are expressed in a various types of cell from a number of human tissues or organs. For example, human *CCKBR* is expressed in cells of the pancreas, stomach, skin, nervous system, human *GLP2R* is expressed in cells of the stomach, intestines, pancreas, connective tissue, breast, liver and lungs. The insulin receptor gene (*INSR*) is one of the ubiquitously expressed genes [121]. Most of the organs and tissues where the genes in question are expressed are not involved in appetite regulation, indicating that the encoded proteins can control biological processes other than appetite regulation. Thus, it can be assumed that the ancestral forms of *CCKBR, GLP2R, INSR* which functioned from the early stages of evolution, were involved in appetite regulation later, at the time of formation of specialized brain structures.

It should be noted that three genes associated with developmental processes were found in the group of human genes of ancient origin (PAI = 1 or 2) (Table S6). These genes are *NTRK2, OPRD1,* and *INSR*. Notably, two human genes (*NTRK2* and *INSR*) encode receptors with tyrosine kinase activity. In unicellular organisms (bacteria and yeast), proteins with tyrosine kinase activity also play an essential role in vital cellular processes. For example, it is known that bacterial tyrosine kinases regulate proteins involved in such processes as translation, carbohydrate metabolism, and DNA replication [122]. In yeast, proteins with tyrosine kinase activity regulate the activity of proteins involved in cellular carbohydrate processes (glycolysis/gluconeogenesis), translation, protein folding, ion homeostasis, and nucleotide and amino acid metabolism [123]. Of particular interest is the finding that in mice, neurotrophic receptor tyrosine kinase 2 (NTRK2) participates in tooth development: this receptor promotes epithelial invagination during early tooth development [75].

#### 4.4.3 Genes whose ancestral forms were formed at the stage of metazoa divergence (PAI = 3)

At this evolutionary stage, multicellular organisms emerged, having a life cycle that included an embryonic stage of development [124], 125]. According to our analysis, among the four genes with PAI value equal to 3 (Table S6), three genes (*ADIPOR1, BMPR1A*, and *EPHA3*) are associated with development processes. Moreover, two genes (*EPHA3* and *BMPR1A*) are involved in the regulation of embryogenesis.

*EPHA3* encodes a receptor whose ligands are membrane-bound proteins from ephrin family ligands. These ligands reside on a membrane of the adjacent cells. The interaction between membrane receptors from the EPH family with ephrins activates signal transduction pathways in neighboring contacting cells. Eph-receptors and ephrin proteins fulfill specialized roles in patterning the vertebrate body plan. Eph-receptors and ephrin proteins are involved in early embryonic cell movements, that establish the germ layers, define tissue boundaries and pathfinding of axons [126]. The functions of EPHA3 also include the regulation of heart [56] and cerebellum development [57]. According to our analysis, *EPHA3* has DI equal to 0.12. Such a low value of DI indicates that the gene undergoes strong stabilizing selection, and this confirms the function of this gene as a key regulator of embryonic development.

*BMPR1A* encodes bone morphogenetic protein receptor type 1A, which is a receptor for bone morphogenetic proteins (BMP2, BMP4, BMP6 etc.) [127], [128], [129]. In turn, bone morphogenetic proteins (BMPs) play a critical role in embryogenesis. Thus, *BMPR1A* is one of the fundamental regulators of embryogenesis. The DI value for this gene is equal to 0.058, i.e., *BMPR1A* was under strong pressure of stabilizing selection. Mouse embryos lacking *Bmpr1a* fail to gastrulate. In addition, BMP receptor IA is required in the mammalian embryo for endodermal morphogenesis and ectodermal patterning [58]. BMPR1A also maintains palatal epithelial integrity during palatogenesis [59]. Bone morphogenetic proteins (BMPs) regulate bone development and bone homeostasis by activating both Smad-dependent and non-Smad-dependent signaling pathways [130]. So *BMPR1A* is involved in regulation bone mass along with bone composition (mineral-to-matrix ratio) [131].

*ADIPOR1* the third development-associated gene, in this group encodes adiponectin receptor essential for embryogenesis. Combined ADIPOR1/ADIPOR2 deficiency causes embryonic lethality in mice by disrupting sphingosine-1-phosphate signaling, which in turn leads to a failure in membrane homeostasis [132]. In addition, it was shown that in a human embryonic kidney 293 cell model ADIPOR1 can activate ERK1/2 through a Src/Ras-dependent pathway and stimulate cell growth [110].

#### 4.4.4 Genes originating during chordata and craniata divergence (PAI = 4 and 5)

During these evolutionary stages, the chordate body plan and gastrointestinal tract were established. According to paleontological evidence, the formation of the body plan of the chordates occurred during the Cambrian explosion and included the development of the chord, circulatory system and gastrointestinal tract [133]. These structures are discernible on the fossilized remains. According to our data (Figure 3A), a very small proportion (1.6 %), that is, only 305 genes out of 19,504 genes from the set *allCDS_19,504* (all human protein-coding genes) have PAI values equal to 4 or 5. Consequently, we found only one gene (*DRD1*) having phylostratigraphic age corresponding to PAI values equal to 4 or 5 within the *Receptors_80* set (Table S6). *DRD1* encodes dopamine receptor D1, which mediates the anorexigenic effect of this neurotransmitter [111]. Upon dopamine binding, DRD1 receptor activates adenylate cyclase, elevating cAMP level in the cell [134]. As noted above, *DRD1* was subjected to stabilizing selection (DI = 0.026). It should be noted that the other dopamine receptor, DRD2, also involved in appetite regulation (encoded by *DRD2*), on the contrary, mediates the orexigenic effect of dopamine and acts on adenylate cyclase in the opposite way (suppresses the activity of adenylate cyclase) [134]. The formation of the ancestral form of the DRD2 is dated by the phylostratigraphic age index to a later evolutionary stage – the stage of Vertebrata divergence (PAI = 6).

#### 4.4.5 Genes whose ancestral forms were formed at the stages of vertebrata divergence (PAI = 6)

At this evolutionary stage the development of vertebrae, cranium, and facial structures including jaws and teeth, facilitating food acquisition was taking place. Analyzing PAI values of genes (Table S6) we have found that the set of genes encoding cell surface receptors controlling appetite (*Receptors_80*) showed an elevated proportion of genes with the same phylostratigraphic age (PAI = 6, the stage of Vertebrata divergence) (Figure 3A, Table S7). These 37 genes likely evolved synchronously. Vertebrata divergence thus represents a critical period for appetite regulation evolution, coinciding with: (1) brain emergence as a discrete organ [135], and (2) development of jaws and teeth for efficient feeding.

We found that most of these genes with PAI equal to six (35 out of 37) encode GPCRs, consistent with this superfamily’s established role in transducing hormonal, neurotransmitter, and sensory signals [33]. Thus, among genes encoding GPCRs with a PAI of 6 (the stage of Vertebrata divergence) there are genes encoding receptors for ghrelin (*GHSR*), alpha-melanocyte stimulating hormone (*MC3R* and *MC4R*) and neuropeptide Y (*NPY1R, NPY2R, NPY4R, NPY5R*). Ghrelin is secreted from endocrine X/A-like cells of the stomach and is known to exert an orexigenic effect as well as stimulation of gastrointestinal motility [106]. Alpha-melanocyte stimulating hormone and neuropeptide Y are signalling molecules secreted by neurons of the arcuate nucleus of the hypothalamus. This brain structure is known as a central feeding behavior regulator [3], 8].

According to our analysis, nine out of the 37 genes with PAI value equal to 6 (*NPY1R, GHSR, CNR1, NPY2R, DRD2, NPY5R, AVPR1A, CRHR1, LGR4*), are associated with developmental processes (Table S5). Among them, four genes (*LGR4, NPY1R*, and *NPY5R*) are involved in the regulation of embryogenesis in humans or related species.

*LGR4* encodes leucine-rich repeat containing G protein-coupled receptor 4. LGR4 mediates the effects of R-spondins. R-spondins are secreted proteins that have pleiotropic functions in development and stem cell growth. LGR4 activation by R-spondins potentiates Wnt/β-catenin signaling by enhancing Wnt-induced LRP6 phosphorylation [60]. Thus, LGR4 is an essential regulator of multiple developmental processes, both at prenatal and postnatal stages of ontogenesis [61], including bone differentiation and mineralization at prenatal and postnatal stages [136], definitive erythropoiesis at midgestation [137], development of heart, liver, kidney, intestine, gonads, prostate, uterine, and oviducts [61]. Notably, LGR4 affects jaws and teeth development [72], [73], [74].

*NPY1R, NPY2R*, and *NPY5R* encode receptors for neuropeptide Y (NPY). NPY and its Y1 and Y5 receptors are expressed in undifferentiated human embryonic stem cells. Inhibition of NPY signalling using either the selective NPY Y1 or Y5 receptor antagonist reduces the maintenance of self-renewal and proliferation of undifferentiated human embryonic stem cells [20]. Central NPY Y2 receptors are involved in the regulation of bone formation, since selective deletion of hypothalamic Y2 receptors in mature conditional Y2 knockout mice stimulated osteoblast activity and increased the rate of bone mineralization and formation [138]. NPY R1 and NPY R2 receptors mediate the neuroprotective and neurogenic effects effect of NPY in a mouse model of Alzheimer’s Disease [21].

*GHSR* (growth hormone secretagogue receptor), CNR1 (cannabinoid receptor 1) and DRD2 (dopamine receptor D2) regulate growth processes by modulating pituitary growth hormone secretion, as demonstrated in mice [19], 108], 139], 140].

*CRHR1* encodes corticotropin releasing hormone receptor 1. In mice this receptor mediates neuritogenic effect of corticotropin releasing hormone; activation of CRHR1 promoted growth arrest and neurite elongation in mouse hippocampal neuronal cell line HT22 [71].

*AVPR1A* encodes arginine-vasopressin receptor and may influence physiological stress regulation and blood pressure [141]. In rats treated with the AVPR1A antagonist it was shown that this receptor contributes to NF-kappaB and cyclin (D1 and A) activation by vasopressin, to hepatocyte progression in the cell cycle, and to liver mass restoration after partial hepatectomy [142].

#### 4.4.6 Genes whose ancestral forms emerged at the stages of Euteleostomi and Mammalia divergence (PAI = 7 or 8)

At these evolutionary stages, key developments include skeletal ossification, the acquisition of thermogenesis and full-fledged chewing function, and the formation of the immune system. Additionally, the bone system acts as a limiting factor in growth and development.

We found that 18 of the 80 genes from the *Receptors_80* set have ancestral forms that can be dated to these stages (Table S6). It is noteworthy that this group is enriched with genes encoding receptors outside the GPCR superfamily. Six out of 18 genes (which is 33 %) with PAI equal to 7 or 8 do not belong to GPCR superfamily. These six genes are: *TLR2, LEPR, TLR4, GHR, IL1R1, GFRAL* This observation aligns with a distinct feature of the *app_not_GPCR_13* set: analysis of PAI distribution reveals an enrichment of genes with PAI = 6 or 7 (the stages of Euteleostomi and Mammalia divergence) (Figure 4).

Five out of six genes from the *app_not_GPCR_13* set having PAI = 6 or 7 (Table S6) are related to the regulation of immunity. These are (1) *IL1R1*, encoding a mediator of cytokine-induced immune and inflammatory responses; (2) *TLR2* and *TLR4*, encoding proteins from the Toll-like receptor family; (3) *LEPR* and *GHR* encoding proteins from the type I cytokine receptor family.

Another gene with PAI = 7, *CMKLR1* (chemerin chemokine-like receptor 1), belongs to the GPCR superfamily (*appGPCR_67* set) and, is also implicated in immune function, particularly inflammatory responses [143]).

Identification of these five genes from the *app_not_GPCR_13* set, as well as *CMKLR1*, among the subset of relatively “young” genes is well consistent with the already established ideas that the formation of the adaptive immunity occurred relatively recently in the course of evolution [144]. It can be assumed that the evolutionary motivation for the involvement of innate immune system genes in appetite regulation consisted in (a) suppressing appetite upon consuming unsuitable food that causes allergic reactions; (b) regulating the population size by reducing the viability of diseased individuals.

According to our analysis, out of 18 genes with PAI equal to 7 or 8, five genes (*TLR4, AGTR2, LEPR, GHR*, and *BDKRB1*) are associated with developmental processes. Notably, two of them (*TLR4*, and *AGTR2*) are directly involved in the regulation of embryogenesis in humans or closely related species.

*TLR4* encodes Toll-like receptor 4, which plays essential role during embryo implantation and morphogenesis. In mouse models, this receptor mediates inflammation-like response in the pre-implantation uterus that induces generation of regulatory T cells. These T cells enable mouse embryo implantation and support robust pregnancy tolerance, ensuring optimal fetal growth and survival [62]. In addition, bacterial infection of maternal tissues can activate TLR4 via lipopolysaccharides. In turn, activated TLR4 may induce TNF-α, which acts on the placenta and/or fetus, initiating placenta necrosis in mice [145]. TLR4 can activate NF-kB signaling pathway – a key regulator of embryonic development, including hematopoietic stem cells (HSCs) specification [63] and epididymal embryonic development [64].

*AGTR2* encodes type 2 angiotensin II receptor. Its receptor expression during the fetal vasculogenesis can influence the growth phenotype of vascular smooth muscle cells via the modulation of ERK cascade [65].

*GHR* encodes receptor for growth hormone which promotes postnatal human growth [146].

*LEPR* encodes leptin receptor which regulates neurogenesis in mice among other functions. It was shown that leptin-deficient (LepOb) mice exhibit altered brain volume, reduced neurogenesis and memory impairment. Similar effects were observed in animals that do not express the LEPR [68].

*BDKRB1* encodes bradykinin receptor B1, mediating effects of pro-inflammatory peptide bradykinin. Bradykinin may activate migration and invasion of human glioblastoma cells [147]. BDKRB1 contributes to the development of doxorubicin-induced cardiomyopathy in mice [148].

## 5 Conclusions

Here we present a compilation of genes, encoding cell surface receptors, whose orthologs regulated food intake in model animal species.

Analysis of the phylostratigraphic age of genes encoding cell surface receptors and regulating appetite showed that their evolutionary origins are distributed in the range from the root of the phylogenetic tree (*Cellular organisms*) to the stage of Mammalia divergence. A significant portion of these genes (46 %), most of which belong to the GPCR superfamily, have a phylostratigraphic age corresponding to the stage of Vertebrata divergence. Notably, this period involved key innovations: the emergence of the brain as a distinct organ and the development of jaws. These adaptations enabled new feeding strategies, including foraging, eating, digestion, and systemic nutrient distribution. Consequently, this required the coordinated evolution of genes encoding appetite-controlling receptors with each other and other organismal systems. In this context, our finding that a significant portion (46 %) of the genes encoding cell surface receptors involved in appetite regulation, but not belonging to the GPCR subfamily, have a phylostratigraphic age corresponding to the stages of Euteleostomi or Mammalia divergence, confirms this idea. A significant proportion of these genes are associated with the immune system.

Analyzing the evolutionary characteristics of genes based on the divergence index (DI), we found that the genes of appetite-regulating receptors undergo stronger stabilizing selection than all human protein-coding genes. It was also revealed that a subgroup of appetite-regulating receptor genes with a low DI (less than or equal to 0.25), i.e., those undergoing the strongest stabilizing selection, is enriched with genes associated with developmental processes.

We identified genes with extreme characteristics. It can be assumed that more “ancient” genes (having low PAI value) and genes undergoing stabilizing selection (having low DI value) may be involved in a greater number of important biological processes. Consequently, selecting such genes as pharmacological targets could increase the risk of off-target effects. The evolutionary patterns we observed in appetite-regulating receptors using PAI and DI metrics provide a foundation for further analysis of this gene network.

## Supplementary Material

Supplementary Material Details

Supplementary Material Details

## References

[1] Hileti D, Demetriou CA, Iasonides MC, Pipis S, Mahmood A, Lanigan J (2023). Weight gain in early infancy impacts appetite regulation in the first year of life. A prospective study of infants living in Cyprus. J Nutr.

[2] El-Haddad MA, Desai M, Gayle D, Ross MG (2004). Utero development of fetal thirst and appetite: potential for programming. J Soc Gynecol Invest.

[3] Yeo GS, Heisler LK (2012). Unraveling the brain regulation of appetite: lessons from genetics. Nat Neurosci.

[4] Kaidar-Person O, Bar-Sela G, Person B (2011). The two major epidemics of the twenty-first century: obesity and cancer. Obes Surg.

[5] Olszewski PK, Cedernaes J, Olsson F, Levine AS, Schiöth HB (2008). Analysis of the network of feeding neuroregulators using the Allen Brain Atlas. Neurosci Biobehav Rev.

[6] Tremblay A, Bellisle F (2015). Nutrients, satiety, and control of energy intake. Appl Physiol Nutr Metabol.

[7] Klockars A, Levine AS, Olszewski PK (2019). Hypothalamic integration of the endocrine signaling related to food intake. Curr Top Behav Neurosci.

[8] Heisler LK, Lam DD (2017). An appetite for life: brain regulation of hunger and satiety. Curr Opin Pharmacol.

[9] Ahn BH, Kim M, Kim S-Y (2022). Brain circuits for promoting homeostatic and non-homeostatic appetites. Exp Mol Med.

[10] Rossi MA, Stuber GD (2018). Overlapping brain circuits for homeostatic and hedonic feeding. Cell Metab.

[11] Fulton S (2010). Appetite and reward. Front Neuroendocrinol.

[12] Alonso-Alonso M, Woods SC, Pelchat M, Grigson PS, Stice E, Farooqi S (2015). Food reward system: current perspectives and future research needs. Nutr Rev.

[13] Morales I, Berridge KC (2020). ‘Liking’ and ‘wanting’ in eating and food reward: brain mechanisms and clinical implications. Physiol Behav.

[14] Maniam J, Morris MJ (2012). The link between stress and feeding behaviour. Neuropharmacology.

[15] López-Gambero AJ, Martínez F, Salazar K, Cifuentes M, Nualart F (2019). Brain glucose-sensing mechanism and energy homeostasis. Mol Neurobiol.

[16] Marler KJ, Becker-Barroso E, Martínez A, Llovera M, Wentzel C, Poopalasundaram S (2008). A TrkB/EphrinA interaction controls retinal axon branching and synaptogenesis. J Neurosci.

[17] Holt LM, Hernandez RD, Pacheco NL, Torres Ceja B, Hossain M, Olsen ML (2019). Astrocyte morphogenesis is dependent on BDNF signaling via astrocytic TrkB.T1. eLife.

[18] Vanevski F, Xu B (2013). Molecular and neural bases underlying roles of BDNF in the control of body weight. Front Neurosci.

[19] Takaya K, Ariyasu H, Kanamoto N, Iwakura H, Yoshimoto A, Harada M (2000). Ghrelin strongly stimulates growth hormone release in humans. J Clin Endocrinol Metab.

[20] Son MY, Kim MJ, Yu K, Koo DB, Cho YS (2011). Involvement of neuropeptide Y and its Y1 and Y5 receptors in maintaining self-renewal and proliferation of human embryonic stem cells. J Cell Mol Med.

[21] Spencer B, Potkar R, Metcalf J, Thrin I, Adame A, Rockenstein E (2016). Systemic central nervous system (CNS)-targeted delivery of neuropeptide Y (NPY) reduces neurodegeneration and increases neural precursor cell proliferation in a mouse model of Alzheimer disease. J Biol Chem.

[22] Grossberg AJ, Scarlett JM, Marks DL (2010). Hypothalamic mechanisms in cachexia. Physiol Behav.

[23] Ignatieva EV, Afonnikov DA, Rogaev EI, Kolchanov NA (2014). Human genes controlling feeding behavior or body mass and their functional and genomic characteristics: a review. Vavilov J Genet Breed.

[24] Ignatieva EV, Afonnikov DA, Saik OV, Rogaev EI, Kolchanov NA (2016). A compendium of human genes regulating feeding behavior and body weight, its functional characterization and identification of GWAS genes involved in brain-specific PPI network. BMC Genet.

[25] Piper NBC, Whitfield EA, Stewart GD, Xu X, Furness SGB (2022). Targeting appetite and satiety in diabetes and obesity, via G protein-coupled receptors. Biochem Pharmacol.

[26] Jamaluddin A, Gorvin CM (2023). RISING STARS: targeting G protein-coupled receptors to regulate energy homeostasis. J Mol Endocrinol.

[27] Bjarnadóttir TK, Gloriam DE, Hellstrand SH, Kristiansson H, Fredriksson R, Schiöth HB (2006). Comprehensive repertoire and phylogenetic analysis of the G protein-coupled receptors in human and mouse. Genomics.

[28] Bausch-Fluck D, Goldmann U, Müller S, van Oostrum M, Müller M, Schubert OT (2018). The in silico human surfaceome. Proc Natl Acad Sci.

[29] Ignatieva EV, Lashin SA, Mustafin ZS, Kolchanov NA (2023). Evolution of human genes encoding cell surface receptors involved in the regulation of appetite: an analysis based on the phylostratigraphic age and divergence indexes. Vavilovskii Zhurnal Genet Selektsii.

[30] Mustafin ZS, Lashin SA, Matushkin YG (2021). Phylostratigraphic analysis of gene networks of human diseases. Vavilovskii Zhurnal Genet Selektsii.

[31] Mustafin ZS, Lashin SA, Matushkin YG, Gunbin KV, Afonnikov DA (2017). Orthoscape: a cytoscape application for grouping and visualization KEGG based gene networks by taxonomy and homology principles. BMC Bioinf.

[32] Kooistra AJ, Mordalski S, Pándy-Szekeres G, Esguerra M, Mamyrbekov A, Munk C (2021). GPCRdb in 2021: integrating GPCR sequence, structure and function. Nucleic Acids Res.

[33] Pándy-Szekeres G, Caroli J, Mamyrbekov A, Kermani AA, Keserű GM, Kooistra AJ (2023). GPCRdb in 2023: state-specific structure models using AlphaFold2 and new ligand resources. Nucleic Acids Res.

[34] Wells A, Kopp N, Xu X, O’Brien DR, Yang W, Nehorai A (2015). The anatomical distribution of genetic associations. Nucleic Acids Res.

[35] Consortium GTE, Deluca DS, Segrè AV, Sullivan TJ, Young TR, Gelfand ET (2015). The genotype-tissue expression (GTEx) pilot analysis: multitissue gene regulation in humans. Science.

[36] Sherman BT, Hao M, Qiu J, Jiao X, Baseler MW, Lane HC (2022). DAVID: a web server for functional enrichment analysis and functional annotation of gene lists (2021 update). Nucleic Acids Res.

[37] Bell EA, Boehnke P, Harrison TM, Mao WL (2015). Potentially biogenic carbon preserved in a 4.1 billion-year-old zircon. Proc Natl Acad Sci U S A.

[38] Leander BS (2020). Predatory protists. Curr Biol.

[39] Maloof AC, Rose CV, Beach R, Samuels BM, Calmet CC, Erwin DH (2010). Possible animal-body fossils in pre-marinoan limestones from South Australia. Nat Geosci.

[40] Maloof AC, Porter SM, Moore JL, Dudas FO, Bowring SA, Higgins JA (2010). The earliest Cambrian record of animals and ocean geochemical change. Geol Soc Am Bull.

[41] Shu D-G, Luo H-L, Conway Morris S, Zhang X-L, Hu S-X, Chen L (1999). Lower Cambrian vertebrates from South China. Nature.

[42] Carroll RL (2008). The origin of higher clades. Osteology, myology, phylogeny and evolution of bony fishes and the rise of tetrapods. Rui Diogo. Integr Comp Biol.

[43] Datta PM (2005). Earliest mammal with transversely expanded upper molar from the Late Triassic (Carnian) Tiki Formation, South Rewa Gondwana Basin, India. J Vertebr Paleontol.

[44] Luo ZX, Yuan CX, Meng QJ, Ji QA (2011). Jurassic eutherian mammal and divergence of marsupials and placentals. Nature.

[45] Kumar V, Hallstrom BM, Janke A (2013). Coalescent-based genome analyses resolve the early branches of the euarchontoglires. PLoS One.

[46] Chatterjee HJ, Ho SY, Barnes I, Groves C (2009). Estimating the phylogeny and divergence times of primates using a supermatrix approach. BMC Evol Biol.

[47] Dunn RH, Rose KD, Rana RS, Kumar K, Sahni A, Smith T (2016). New euprimate postcrania from the early Eocene of Gujarat, India, and the strepsirrhine-haplorhine divergence. J Hum Evol.

[48] Harrison T. (2013). Catarrhine origins. A companion to paleoanthropology.

[49] Hey J (2005). The ancestor’s tale: a pilgrimage to the dawn of evolution. J Clin Invest.

[50] Schrenk F, Kullmer O, Bromage T, Henke W, Tattersall I (2014). The earliest putative homo fossils. Handbook of paleoanthropology.

[51] Scerri EML, Thomas MG, Manica A, Gunz P, Stock JT, Stringer C (2018). Did our species evolve in subdivided populations across Africa, and why does it matter?. Trends Ecol Evol.

[52] Ruiz de Azua I, Martin-Garcia E, Domingo-Rodriguez L, Aparisi Rey A, Pascual Cuadrado D, Islami L (2021). Cannabinoid CB1 receptor in dorsal telencephalic glutamatergic neurons drives overconsumption of palatable food and obesity. Neuropsychopharmacology.

[53] Wardman JH, Gomes I, Bobeck EN, Stockert JA, Kapoor A, Bisignano P (2016). Identification of a small-molecule ligand that activates the neuropeptide receptor GPR171 and increases food intake. Sci Signal.

[54] Ohinata K, Fujiwara Y, Fukumoto S, Iwai M, Horiuchi M, Yoshikawa M (2008). Angiotensin II and III suppress food intake via angiotensin AT(2) receptor and prostaglandin EP(4) receptor in mice. FEBS Lett.

[55] Stein LM, McGrath LE, Lhamo R, Koch-Laskowski K, Fortin SM, Skarbaliene J (2021). The long-acting amylin/calcitonin receptor agonist ZP5461 suppresses food intake and body weight in male rats. Am J Physiol Regul Integr Comp Physiol.

[56] Stephen LJ, Fawkes AL, Verhoeve A, Lemke G, Brown A (2007). A critical role for the EphA3 receptor tyrosine kinase in heart development. Dev Biol.

[57] Karam SD, Burrows RC, Logan C, Koblar S, Pasquale EB, Bothwell M (2000). Eph receptors and ephrins in the developing chick cerebellum: relationship to sagittal patterning and granule cell migration. J Neurosci.

[58] Davis S, Miura S, Hill C, Mishina Y, Klingensmith J (2004). BMP receptor IA is required in the mammalian embryo for endodermal morphogenesis and ectodermal patterning. Dev Biol.

[59] He F, Xiong W, Wang Y, Matsui M, Yu X, Chai Y (2010). Modulation of BMP signaling by noggin is required for the maintenance of palatal epithelial integrity during palatogenesis. Dev Biol.

[60] Carmon KS, Gong X, Lin Q, Thomas A, Liu Q (2011). R-spondins function as ligands of the orphan receptors LGR4 and LGR5 to regulate Wnt/beta-catenin signaling. Proc Natl Acad Sci U S A.

[61] Yang L, Wang J, Gong X, Fan Q, Yang X, Cui Y (2022). Emerging roles for LGR4 in organ development, energy metabolism and carcinogenesis. Front Genet.

[62] Chan HY, Moldenhauer LM, Groome HM, Schjenken JE, Robertson SA (2021). Toll-like receptor-4 null mutation causes fetal loss and fetal growth restriction associated with impaired maternal immune tolerance in mice. Sci Rep.

[63] He Q, Zhang C, Wang L, Zhang P, Ma D, Lv J (2015). Inflammatory signaling regulates hematopoietic stem and progenitor cell emergence in vertebrates. Blood.

[64] Ferreira LGA, Nishino FA, Fernandes SG, Ribeiro CM, Hinton BT, Avellar MCW (2022). Epididymal embryonic development harbors TLR4/NFKB signaling pathway as a morphogenetic player. J Reprod Immunol.

[65] Akishita M, Ito M, Lehtonen JY, Daviet L, Dzau VJ, Horiuchi M (1999). Expression of the AT2 receptor developmentally programs extracellular signal-regulated kinase activity and influences fetal vascular growth. J Clin Invest.

[66] Wang Q, Dong X, Lu J, Hu T, Pei G (2020). Constitutive activity of a G protein-coupled receptor, DRD1, contributes to human cerebral organoid formation. Stem Cells.

[67] Roussel-Gervais A, Sgroi S, Cambet Y, Lemeille S, Seredenina T, Krause KH (2023). Genetic knockout of NTRK2 by CRISPR/Cas9 decreases neurogenesis and favors glial progenitors during differentiation of neural progenitor stem cells. Front Cell Neurosci.

[68] Fernandes C, Forny-Germano L, Andrade MM, Lyra E, Silva NM, Ramos-Lobo AM (2024). Leptin receptor reactivation restores brain function in early-life Lepr-deficient mice. Brain.

[69] Lin YT, Chen CC, Huang CC, Nishimori K, Hsu KS (2017). Oxytocin stimulates hippocampal neurogenesis via oxytocin receptor expressed in CA3 pyramidal neurons. Nat Commun.

[70] Pekarek BT, Kochukov M, Lozzi B, Wu T, Hunt PJ, Tepe B (2022). Oxytocin signaling is necessary for synaptic maturation of adult-born neurons. Genes Dev.

[71] Inda C, Bonfiglio JJ, Dos Santos Claro PA, Senin SA, Armando NG, Deussing JM (2017). cAMP-dependent cell differentiation triggered by activated CRHR1 in hippocampal neuronal cells. Sci Rep.

[72] Yamakami Y, Kohashi K, Oyama K, Mohri Y, Hidema S, Nishimori K (2016). LGR4 is required for sequential molar development. Biochem Biophys Rep.

[73] Matsuike R, Tanaka H, Nakai K, Kanda M, Nagasaki M, Murakami F (2018). Continuous application of compressive force induces fusion of osteoclast-like RAW264.7 cells via upregulation of RANK and downregulation of LGR4. Life Sci.

[74] Arima M, Hasegawa D, Yoshida S, Mitarai H, Tomokiyo A, Hamano S (2019). R-spondin 2 promotes osteoblastic differentiation of immature human periodontal ligament cells through the Wnt/β-catenin signaling pathway. J Periodontal Res.

[75] Ye Q, Bhojwani A, Hu JK (2022). Understanding the development of oral epithelial organs through single cell transcriptomic analysis. Development.

[76] Domazet-Lošo T, Brajković J, Tautz D (2007). A phylostratigraphy approach to uncover the genomic history of major adaptations in metazoan lineages. Trends Genet.

[77] Barrera-Redondo J, Lotharukpong JS, Drost HG, Coelho SM (2023). Uncovering gene-family founder events during major evolutionary transitions in animals, plants and fungi using GenEra. Genome Biol.

[78] Yang D, Zhou Q, Labroska V, Qin S, Darbalaei S, Wu Y (2021). G protein-coupled receptors: structure- and function-based drug discovery. Signal Transduction Targeted Ther.

[79] Johnson AW (2013). Eating beyond metabolic need: how environmental cues influence feeding behavior. Trends Neurosci.

[80] Rebello CJ, Greenway FL (2016). Reward-induced eating: therapeutic approaches to addressing food cravings. Adv Ther.

[81] Tomé D, Schwarz J, Darcel N, Fromentin G (2009). Protein, amino acids, vagus nerve signaling, and the brain. Am J Clin Nutr.

[82] Holtmann G, Talley NJ (2014). The stomach-brain axis. Best Pract Res Clin Gastroenterol.

[83] Spetter MS, de Graaf C, Mars M, Viergever MA, Smeets PA (2014). The sum of its parts--effects of gastric distention, nutrient content and sensory stimulation on brain activation. PLoS One.

[84] Braden A, Barnhart WR, Kalantzis M, Redondo R, Dauber A, Anderson L (2023). Eating when depressed, anxious, bored, or happy: an examination in treatment-seeking adults with overweight/obesity. Appetite.

[85] Braden A, Musher-Eizenman D, Watford T, Emley E (2018). Eating when depressed, anxious, bored, or happy: are emotional eating types associated with unique psychological and physical health correlates?. Appetite.

[86] Siegal E, Hooker SK, Isojunno S, Miller PJO (2022). Beaked whales and state-dependent decision-making: how does body condition affect the trade-off between foraging and predator avoidance?. Proc Biol Sci.

[87] Hadjieconomou D, King G, Gaspar P, Mineo A, Blackie L, Ameku T (2020). Enteric neurons increase maternal food intake during reproduction. Nature.

[88] Lindén A, Hansen S, Bednar I, Forsberg G, Södersten P, Uvnäs-Moberg K (1987). Sexual activity increases plasma concentrations of cholecystokinin octapeptide and offsets hunger in male rats. J Endocrinol.

[89] McHill AW, Hull JT, Klerman EB (2022). Chronic circadian disruption and sleep restriction influence subjective hunger, appetite, and food preference. Nutrients.

[90] Johnson ML, Saffrey MJ, Taylor VJ (2019). Gastrointestinal capacity, gut hormones and appetite change during rat pregnancy and lactation. Reproduction.

[91] Patil CL, Abrams ET, Steinmetz AR, Young SL (2012). Appetite sensations and nausea and vomiting in pregnancy: an overview of the explanations. Ecol Food Nutr.

[92] Hirschberg AL (2012). Sex hormones, appetite and eating behaviour in women. Maturitas.

[93] Chen D, Liu X, Zhang W, Shi Y (2012). Targeted inactivation of GPR26 leads to hyperphagia and adiposity by activating AMPK in the hypothalamus. PLoS One.

[94] Zhang LL, Wang JJ, Liu Y, Lu XB, Kuang Y, Wan YH (2011). GPR26-deficient mice display increased anxiety- and depression-like behaviors accompanied by reduced phosphorylated cyclic AMP responsive element-binding protein level in central amygdala. Neuroscience.

[95] Jones PG, Nawoschik SP, Sreekumar K, Uveges AJ, Tseng E, Zhang L (2007). Tissue distribution and functional analyses of the constitutively active orphan G protein coupled receptors, GPR26 and GPR78. Biochim Biophys Acta.

[96] Salim K, Fenton T, Bacha J, Urien-Rodriguez H, Bonnert T, Skynner HA (2002). Oligomerization of G-protein-coupled receptors shown by selective co-immunoprecipitation. J Biol Chem.

[97] Hashimoto K (2014). Activation of sigma-1 receptor chaperone in the treatment of neuropsychiatric diseases and its clinical implication. J Pharmacol Sci.

[98] Schmidt HR, Zheng S, Gurpinar E, Koehl A, Manglik A, Kruse AC (2016). Crystal structure of the human σ1 receptor. Nature.

[99] Ashrafi K, Chang FY, Watts JL, Fraser AG, Kamath RS, Ahringer J (2003). Genome-wide RNAi analysis of *Caenorhabditis elegans* fat regulatory genes. Nature.

[100] Grosse J, Heffron H, Burling K, Akhter Hossain M, Habib AM, Rogers GJ (2014). Insulin-like peptide 5 is an orexigenic gastrointestinal hormone. Proc Natl Acad Sci U S A.

[101] Cook C, Nunn N, Worth AA, Bechtold DA, Suter T, Gackeheimer S (2022). The hypothalamic RFamide, QRFP, increases feeding and locomotor activity: the role of Gpr103 and orexin receptors. PLoS One.

[102] Herraiz C, Martínez-Vicente I, Maresca V (2021). The α-melanocyte-stimulating hormone/melanocortin-1 receptor interaction: a driver of pleiotropic effects beyond pigmentation. Pigm Cell Melanoma Res.

[103] Singh M, Mukhopadhyay K (2014). Alpha-melanocyte stimulating hormone: an emerging anti-inflammatory antimicrobial peptide. BioMed Res Int.

[104] Lee HJ, Macbeth AH, Pagani JH, Young WS (2009). Oxytocin: the great facilitator of life. Prog Neurobiol.

[105] Nishimori K, Takayanagi Y, Yoshida M, Kasahara Y, Young LJ, Kawamata M (2008). New aspects of oxytocin receptor function revealed by knockout mice: sociosexual behaviour and control of energy balance. Prog Brain Res.

[106] Stengel A, Taché Y (2012). Ghrelin - a pleiotropic hormone secreted from endocrine x/a-like cells of the stomach. Front Neurosci.

[107] Atas U, Erin N, Tazegul G, Elpek GO, Yildirim B (2021). Changes in ghrelin, substance P and vasoactive intestinal peptide levels in the gastroduodenal mucosa of patients with morbid obesity. Neuropeptides.

[108] Al-Massadi O, Gabellieri E, Trujillo ML, Señaris R, Pagotto U, Pasquali R (2010). Peripheral endocannabinoid system-mediated actions of rimonabant on growth hormone secretion are ghrelin-dependent. J Neuroendocrinol.

[109] Coope A, Milanski M, Araújo EP, Tambascia M, Saad MJ, Geloneze B (2008). AdipoR1 mediates the anorexigenic and insulin/leptin-like actions of adiponectin in the hypothalamus. FEBS Lett.

[110] Lee MH, Klein RL, El-Shewy HM, Luttrell DK, Luttrell LM (2008). The adiponectin receptors AdipoR1 and AdipoR2 activate ERK1/2 through a Src/Ras-dependent pathway and stimulate cell growth. Biochemistry.

[111] Martin-Iverson MT, Dourish CT (1988). Role of dopamine D-1 and D-2 receptor subtypes in mediating dopamine agonist effects on food consumption in rats. Psychopharmacology.

[112] Koonin EV (2005). Orthologs, paralogs, and evolutionary genomics. Annu Rev Genet.

[113] Domazet-Loso T, Tautz D (2010). Phylostratigraphic tracking of cancer genes suggests a link to the emergence of multicellularity in metazoa. BMC Biol.

[114] Moyers BA, Zhang J (2017). Further simulations and analyses demonstrate open problems of phylostratigraphy. Genome Biol Evol.

[115] Moyers BA, Zhang J (2015). Phylostratigraphic bias creates spurious patterns of genome evolution. Mol Biol Evol.

[116] Weisman CM, Murray AW, Eddy SR (2020). Many, but not all, lineage-specific genes can be explained by homology detection failure. PLoS Biol.

[117] Chen H, Kent S, Morris MJ (2006). Is the CCK2 receptor essential for normal regulation of body weight and adiposity?. Eur J Neurosci.

[118] Sun H, Meng K, Hou L, Shang L, Yan J (2021). GLP-2 decreases food intake in the dorsomedial hypothalamic nucleus (DMH) through Exendin (9-39) in male Sprague-Dawley (SD) rats. Physiol Behav.

[119] Leibowitz SF, Wortley KE (2004). Hypothalamic control of energy balance: different peptides, different functions. Peptides.

[120] Vithlani M, Hines RM, Zhong P, Terunuma M, Hines DJ, Revilla-Sanchez R (2013). The ability of BDNF to modify neurogenesis and depressive-like behaviors is dependent upon phosphorylation of tyrosine residues 365/367 in the GABA(A)-receptor γ2 subunit. J Neurosci.

[121] Gu J, Dai J, Lu H, Zhao H (2023). Comprehensive analysis of ubiquitously expressed genes in humans from a data-driven perspective. Genom Proteom Bioinf.

[122] Grangeasse C, Nessler S, Mijakovic I (2012). Bacterial tyrosine kinases: evolution, biological function and structural insights. Philos Trans R Soc London Ser B.

[123] Dos Santos SC, Mira NP, Moreira AS, Sá-Correia I (2012). Quantitative- and phospho-proteomic analysis of the yeast response to the tyrosine kinase inhibitor imatinib to pharmacoproteomics-guided drug line extension. OMICS.

[124] Gamulin V, Rinkevich B, Schäcke H, Kruse M, Müller IM, Müller WE (1994). Cell adhesion receptors and nuclear receptors are highly conserved from the lowest metazoa (marine sponges) to vertebrates. Biol Chem Hoppe Seyler.

[125] Müller WE, Müller IM, Gamulin V (1994). On the monophyletic evolution of the metazoa. Braz J Med Biol Res.

[126] Coulthard MG, Duffy S, Down M, Evans B, Power M, Smith F (2002). The role of the Eph-ephrin signalling system in the regulation of developmental patterning. Int J Dev Biol.

[127] Mahlawat P, Ilangovan U, Biswas T, Sun LZ, Hinck AP (2012). Structure of the Alk1 extracellular domain and characterization of its bone morphogenetic protein (BMP) binding properties. Biochemistry.

[128] ten Dijke P, Yamashita H, Sampath TK, Reddi AH, Estevez M, Riddle DL (1994). Identification of type I receptors for osteogenic protein-1 and bone morphogenetic protein-4. J Biol Chem.

[129] Wang CY, Xu Y, Traeger L, Dogan DY, Xiao X, Steinbicker AU (2020). Erythroferrone lowers hepcidin by sequestering BMP2/6 heterodimer from binding to the BMP type I receptor ALK3. Blood.

[130] Niu Z, Zhou Y, Liang M, Su F, Guo Q, Jing J (2024). Crosstalk between ALK3(BMPR1A) deficiency and autophagy signaling mitigates pathological bone loss in osteoporosis. Bone.

[131] Shi C, Mandair GS, Zhang H, Vanrenterghem GG, Ridella R, Takahashi A (2018). Bone morphogenetic protein signaling through ACVR1 and BMPR1A negatively regulates bone mass along with alterations in bone composition. J Struct Biol.

[132] Ruiz M, Devkota R, Panagaki D, Bergh PO, Kaper D, Henricsson M (2022). Sphingosine 1-phosphate mediates adiponectin receptor signaling essential for lipid homeostasis and embryogenesis. Nat Commun.

[133] Erwin DH (1999). The origin of bodyplans. Am Zool.

[134] Neve KA, Seamans JK, Trantham-Davidson H (2004). Dopamine receptor signaling. J Recept Signal Transduction.

[135] Sarnat HB, Netsky MG (2002). When does a ganglion become a brain? Evolutionary origin of the central nervous system. Semin Pediatr Neurol.

[136] Luo J, Zhou W, Zhou X, Li D, Weng J, Yi Z (2009). Regulation of bone formation and remodeling by G-protein-coupled receptor 48. Development.

[137] Song H, Luo J, Luo W, Weng J, Wang Z, Li B (2008). Inactivation of G-protein-coupled receptor 48 (Gpr48/Lgr4) impairs definitive erythropoiesis at midgestation through down-regulation of the ATF4 signaling pathway. J Biol Chem.

[138] Baldock PA, Sainsbury A, Couzens M, Enriquez RF, Thomas GP, Gardiner EM (2002). Hypothalamic Y2 receptors regulate bone formation. J Clin Invest.

[139] Díaz-Torga G, Feierstein C, Libertun C, Gelman D, Kelly MA, Low MJ (2002). Disruption of the D2 dopamine receptor alters GH and IGF-I secretion and causes dwarfism in male mice. Endocrinology.

[140] Noaín D, Pérez-Millán MI, Bello EP, Luque GM, Casas Cordero R, Gelman DM (2013). Central dopamine D2 receptors regulate growth-hormone-dependent body growth and pheromone signaling to conspecific males. J Neurosci.

[141] Nidey N, Bowers K, Ding L, Ji H, Ammerman RT, Yolton K (2023). Neonatal AVPR1a methylation and in-utero exposure to maternal smoking. Toxics.

[142] Nicou A, Serrière V, Prigent S, Boucherie S, Combettes L, Guillon G (2003). Hypothalamic vasopressin release and hepatocyte Ca^2+^ signaling during liver regeneration: an interplay stimulating liver growth and bile flow. FASEB J.

[143] Zhang Z, Ding Y, Li J, Su S (2023). Up-regulation of CMKLR1 in endometriosis and its relationship with inflammatory responses. Histol Histopathol.

[144] Ward AE, Rosenthal BM (2014). Evolutionary responses of innate immunity to adaptive immunity. Infect Genet Evol.

[145] Carpentier PA, Dingman AL, Palmer TD (2011). Placental TNF-α signaling in illness-induced complications of pregnancy. Am J Pathol.

[146] Hwa V (2020). Human growth disorders associated with impaired GH action: defects in STAT5B and JAK2. Mol Cell Endocrinol.

[147] Sun DP, Lee YW, Chen JT, Lin YW, Chen RM (2020). The Bradykinin-BDKRB1 axis regulates aquaporin 4 gene expression and consequential migration and invasion of malignant glioblastoma cells via a Ca2+-MEK1-ERK1/2-NF-κB mechanism. Cancers.

[148] Westermann D, Lettau O, Sobirey M, Riad A, Bader M, Schultheiss HP (2008). Doxorubicin cardiomyopathy-induced inflammation and apoptosis are attenuated by gene deletion of the kinin B1 receptor. Biol Chem.

