# Supplementary material for: Functional and evolutionary characteristics of human genes encoding cell surface receptors involved in the regulation of appetite
Source: J Integr Bioinform. 2026 Jan 8;22(3):20250023. doi: 10.1515/jib-2025-0023 (PMC13066349; doi:10.1515/jib-2025-0023)
Supplement: Supplementary file 2 — Supplementary Material Details [file j_jib-2025-0023_suppl_002.docx]

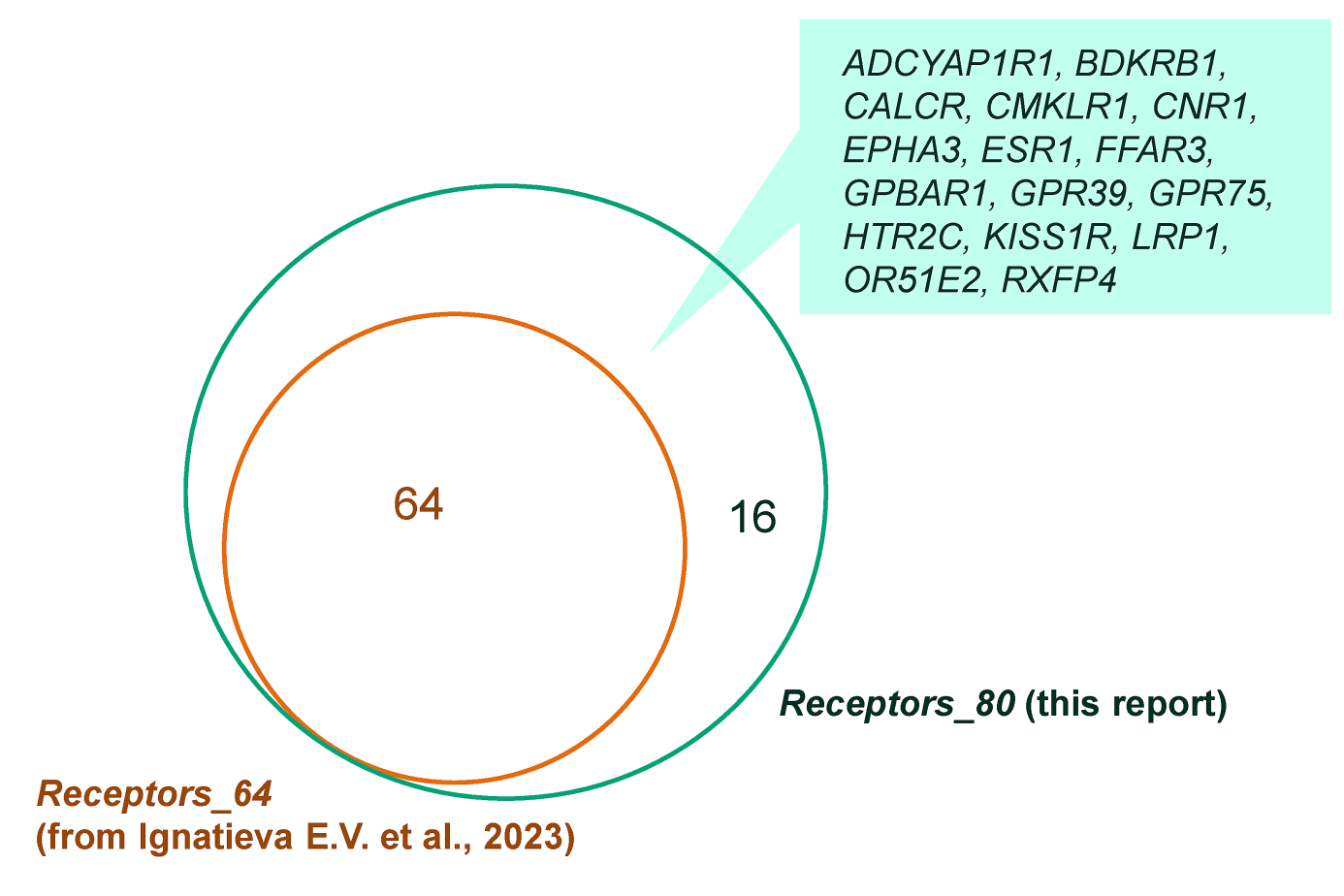


Figure S1. Sets of genes encoding cell surface receptors involved in the regulation of appetite in humans presented in [Ignatieva E.V. et al. Vavilovskii Zhurnal Genet Selektsii. 2023;27(7):829-838] and in the current report
